# Zinc ion rapidly induces toxic, off-pathway amyloid-β oligomers distinct from amyloid-β derived diffusible ligands in Alzheimer’s disease

**DOI:** 10.1038/s41598-018-23122-x

**Published:** 2018-03-19

**Authors:** Ming-Che Lee, Wan-Cheng Yu, Yao-Hsiang Shih, Chun-Yu Chen, Zhong-Hong Guo, Shing-Jong Huang, Jerry C. C. Chan, Yun-Ru Chen

**Affiliations:** 1 Graduate Institute of Life Sciences, National Defense Medical Center, Taipei, Taiwan, R.O.C.; 2 Genomics Research Center, Academia Sinica, Taipei, Taiwan; 3 Department of Chemistry, National Taiwan University, Taipei, Taiwan

## Abstract

Alzheimer’s disease (AD) is the most prevalent neurodegenerative disease in the elderly. Zinc (Zn) ion interacts with the pathogenic hallmark, amyloid-β (Aβ), and is enriched in senile plaques in brain of AD patients. To understand Zn-chelated Aβ (ZnAβ) species, here we systematically characterized ZnAβ aggregates by incubating equimolar Aβ with Zn. We found ZnAβ40 and ZnAβ42 both form spherical oligomers with a diameter of ~12–14 nm composed of reduced β-sheet content. Oligomer assembly examined by analytical ultracentrifugation, hydrophobic exposure by BisANS spectra, and immunoreactivity of ZnAβ and Aβ derived diffusible ligands (ADDLs) are distinct. The site-specific ^13^C labeled solid-state NMR spectra showed that ZnAβ40 adopts β-sheet structure as in Aβ40 fibrils. Interestingly, removal of Zn by EDTA rapidly shifted the equilibrium back to fibrillization pathway with a faster kinetics. Moreover, ZnAβ oligomers have stronger toxicity than ADDLs by cell viability and cytotoxicity assays. The *ex vivo* study showed that ZnAβ oligomers potently inhibited hippocampal LTP in the wild-type C57BL/6JNarl mice. Finally, we demonstrated that ZnAβ oligomers stimulate hippocampal microglia activation in an acute Aβ-injected model. Overall, our study demonstrates that ZnAβ rapidly form toxic and distinct off-pathway oligomers. The finding provides a potential target for AD therapeutic development.

## Introduction

AD is the most common cause of dementia in the elder population after age of 65. All current AD clinical trials have failed due to insignificant beneficial effects or severe adverse effects^1,2^. The failure of clinical trials suggests that the fundamental molecular mechanism of AD pathogenesis is still not fully understood. Aβ, a pathogenic hallmark in AD, is cleaved from amyloid precursor protein by β- and γ-secretases^3,4^. Aβ40 and Aβ42 are the two major isoforms that differ in two additional amino acids in the C- terminus of Aβ^5,6^. Aβ is an intrinsically disordered protein that is prone to aggregate into cross-β-rich fibrils via a nucleation-dependent manner^7^. The classic amyloid fibrillization consists of a nucleation state followed by fibril elongation and a plateau for mature fibril formation. The major cause of AD is considered to associate with assemble of Aβ into oligomers, which impair synaptic function and lead to activation of a cascade of subsequent detrimental events^8,9^. Aβ oligomers are previously referred to heterogeneous intermediates in the aggregation, including various types of species, e.g. prefibrillar oligomer, protofibrils, annular protofibrils, paranuclei, globulomers, amylospheroids, ADDLs, and Aβ56*^9–12^. Despite the intrinsic structural heterogeneity of Aβ oligomeric aggregates, many of their structural features have been unraveled by solid-state NMR^13–16^. Aβ fibrillization can be monitored by thioflavin T (ThT) that emits fluorescence upon chelating to cross-β-stands in amyloid fibrils, however, the oligomer intermediates showed no or low binding to ThT^17^.

Although Aβ40 and Aβ42 are the two major Aβ isoforms, they have distinct properties in structure, aggregation, and toxicity. Freshly prepared Aβ40 was reported to be monomer and Aβ42 adopts rapid equilibrium of monomer and trimer/tetramer^18^. Aβ40 and Aβ42 adopt distinct fibrillization pathways^19,20^. During the aggregation, Aβ42 forms a pentameric/hexameric paranuclei, whereas, Aβ40 undergoes monomer addition^19,20^. Ion mobility mass spectrometry showed Aβ40 assembles through tetramer, whereas, Aβ42 forms tetramer and further forms hexamer that stacks into dodecamer prior to protofibril/fibril formation^20^. The fibril structure of Aβ40 contains two β-strands formed by amino acids 10 to 22 and 30 to 40^21–23^ and Aβ42 fibrils contain multiple β-sheets adopting the so-called LS-shaped structure^24^. Aβ42 is demonstrated more detrimental than Aβ40 *in vitro* and *in vivo*^25,26^ despite the amount of Aβ42 is 10 times less than Aβ40 in cerebrospinal fluids^27^.

Metal ion dyshomeostasis is an existing concept in the AD research^28^. Abnormally high content of metal ions including Zn^2+^, Cu^2+^, Fe^3+^ have been found in senile plaques of postmortem AD brains^29,30^. Many evidences further suggested imbalance of Zn^2+^ or Cu^2+^ homeostasis was linked with AD pathology^31,32^ and association of metal ions with Aβ, especially Cu^2+^ or Zn^2+^ were reported in a series of studies^33–36^. Aβ is known to bind with the metal ions^31,32^. The metal ions, including Zn^2+^ and Cu^2+^, were identified to interact with histidine residues at N-terminal of Aβ^37,38^. The mechanism of interaction of Zn^2+^ with Aβ was reported to share similar coordination with Cu^2+^ by three histidines at residue 6, 13, and 14 of Aβ^39,40^. However, how Zn^2+^ modulates Aβ conformation is still elusive, that may be due to assemblies of ZnAβ were variable and sensitive due to changes of pH, temperature, concentration, and buffer environment. The morphology of ZnAβ was previously shown as fibrils at 37 °C^41^, whereas others discovered that ZnAβ formed amorphous aggregates^42^ or oligomers at room temperature^33,35^. Postmortem brain analysis and *in vivo* model of AD both provided evidences to support that Zn^2+^ might play a role in AD pathologensis^43,44^. Bush *et al*. further developed a Cu/Zn ion chelator, PBT2, to provide beneficial effect for AD^45,46^.

To understand the effect of Zn^2+^ to Aβ40 and Aβ42, here we systematically characterized the oligomer formed by ZnAβ complex. We found Zn^2+^ rapidly induced secondary structure changes and retained Aβ in an oligomer state with higher hydrophobic surface. ZnAβ oligomers have distinct immunoreactivity against several anti-Aβ antibodies and possess higher toxicity than ADDLs. Removal of Zn^2+^ leads to restoration of Aβ fibrillization with a faster kinetics than Aβ alone. In addition, solid-state NMR data suggested that ZnAβ40 oligomers possess β-sheet structure. The electrophysiology result showed that ZnAβ oligomer inhibited hippocampal LTP in the wild-type mice hippocampus slice. In the acute Aβ-injected mice model, increasing of hippocampal microglial activation was found followed by ZnAβ injection.

## Results

### Zn2+ promotes Aβ40 and Aβ42 oligomer formation

**Figure 1:**
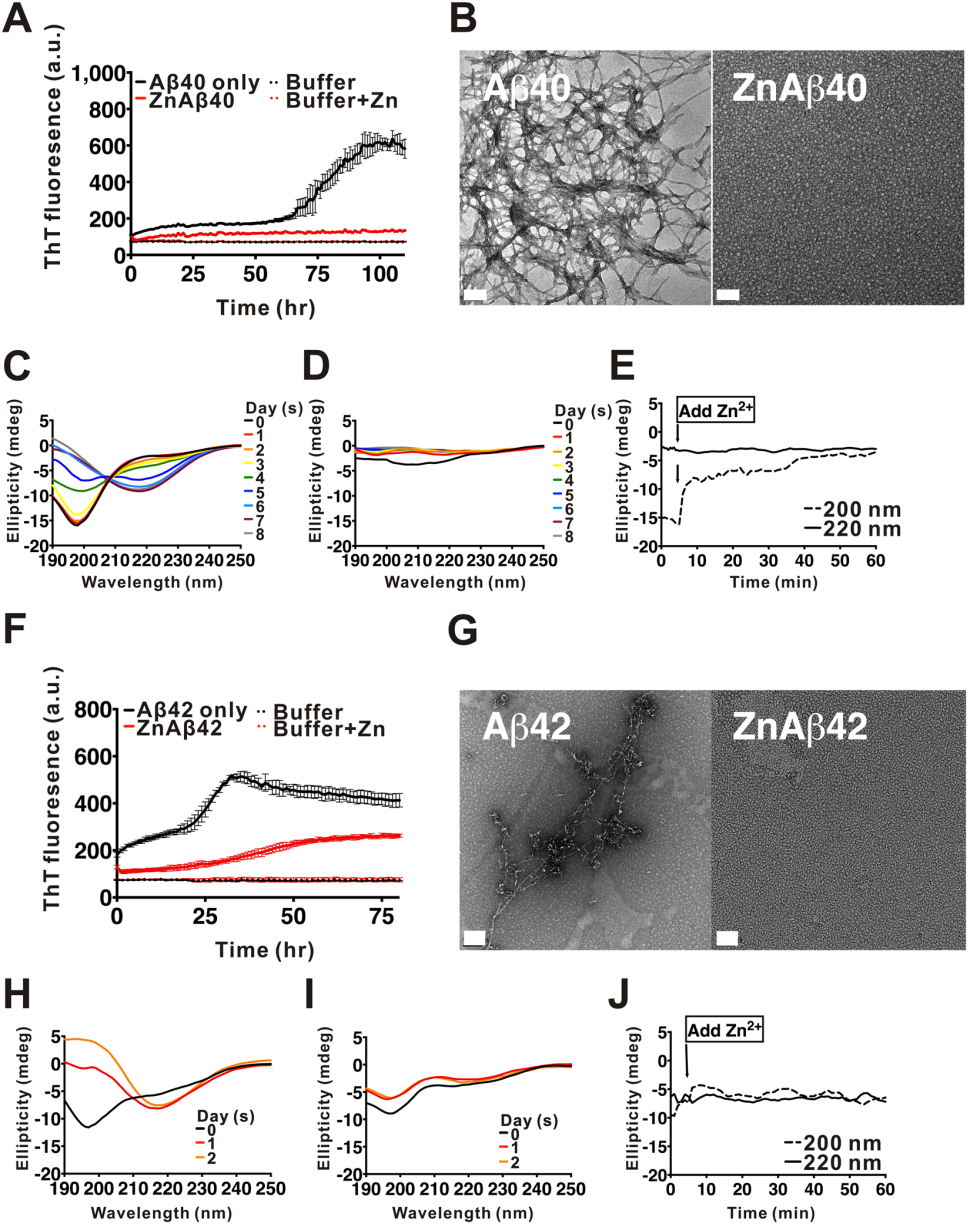
Zn^2+^ promotes Aβ oligomerization. (**A**,**F**) ThT assay of Aβ in the absence or presence of Zn^2+^. Aβ or Aβ42 (F) at 50 µM was incubated with and without equal molar concentration of Zn^2+^. The final ThT concentration in the working solution is 5 µM. ThT fluorescence was monitored at 25 °C. (**B**,**G**) TEM images of the end-point products of Aβ alone or ZnAβ. The scale bars are 100 nm. (**C**–**E**) and (**H**–**J**) Far-UV CD spectra of Aβ40 and Aβ42. The spectra were scanned from 250 to 190 nm at the indicated time. Aβ40 in the absence (**C**) and presence of Zn^2+^ (**D**) after different incubation time are shown. The single wavelength changes of Aβ40 at 200 and 220 nm after addition of Zn^2+^ are shown in panel (**E**). Aβ42 in the absence (**H**) and presence of Zn^2+^ (**I**) were also monitored. The single wavelength changes of Aβ42 at 200 and 220 nm after addition of Zn^2+^ were shown in panel (**J**).

To systematically examine the effect of Zn^2+^ on Aβ aggregation, we incubated equal molar ratio of Zn^2+^ ions with Aβ40 and Aβ42 and examined their fibrillization by ThT assay, the secondary structural changes by far-UV CD, and the epitope changes of the assembly by dot blotting. Aβ40 and Aβ42 were prepared in ammonium hydroxide, lyophilized, and then dissolved in 10 mM Tris-HCl buffer, pH 7.4. Fifty µM Aβ and Zn^2+^ were used in the experiments. In ThT assay, we found that in the absence of Zn^2+^, Aβ40 fibrillization adapted a classic nucleation-dependent response. It showed a lag phase of ~64 hr, and reached maximal ThT fluorescence at ~94 hr (Fig. 1A). Upon addition of equal molar concentration of Zn^2+^ during Aβ40 aggregation, we found the ThT signal was retained without much increase. The result showed that there is lower incidence, if any, of amyloid fibril formation in the presence of Zn^2+^ ions. In the case of Aβ42, Aβ42 alone showed a slow growing phase of ~20 hr, then increased to maximal ThT fluorescence intensity at ~33 hr (Fig. 1F). Whereas, in the presence of Zn^2+^, ThT fluorescence was significantly inhibited similar to the result obtained from Aβ40. The result demonstrated that Zn^2+^ suppresses both Aβ40 and Aβ42 fibrillization. We also examined Aβ40 and Aβ42 fibrillization in various concentrations of Zn^2+^ in ThT assay. Five different concentrations of Zn^2+^ ranging from 10 to 200 µM were added into the 50 µM Aβ solution. The result showed Zn^2+^ suppressed Aβ40 and Aβ42 fibrillization in a dose-dependent manner (Fig. S1). To examine the possible precipitation, we examined the turbidity of the samples for 7 days of incubation by absorbance at 340 nm and also quantified the protein concentration at 280 nm. The samples were turbid especially for the ZnAβ samples indicating large soluble aggregate formation, but no precipitation was observed during the experimental time (Fig. S2). To see whether there is any Aβ aggregation in the presence of Zn^2+^, we imaged the ZnAβ40 and ZnAβ42 aggregates by TEM (Fig. 1B,G, respectively). TEM images showed that in the absence of Zn^2+^ the end-point products of Aβ40 and Aβ42 both formed straight or curvy amyloid fibrils. In contrast, equal molar ratio of Zn^2+^ promoted formation of spherical Aβ species, whose diameters in average were ~14 and ~12 nm for ZnAβ40 and ZnAβ42, respectively, as measured from the TEM images (Fig. S3). Furthermore, we added equal amount of Zn^2+^ into preformed Aβ40 fibrils and studied whether Zn^2+^ is capable to destabilize preformed fibrils by ThT assay and Fourier transform infrared spectra (FTIR). The results showed that the addition of Zn^2+^ ions cannot decrease ThT intensity of Aβ fibrils (Fig. S4A) and does not alter β-sheet structure signals at 1,630 cm^−1^ in FTIR analysis (Fig. S4B). This result suggested that Zn^2+^ only affects Aβ at the early stage.

### Zn^2+^ rapidly changes secondary structures of Aβ40 and Aβ42 and retains Aβ in a less β-conformation

To understand how Zn^2+^ affects the conformation of Aβ, the secondary structure of Aβ40 and Aβ42 with and without Zn^2+^ was examined by far-UV CD spectroscopy for several days (Fig. 1C–E, H–J). On day 0, freshly-prepared A40 showed a random-coil-like spectrum and the conformation gradually transformed to α-helix-like spectra on day 4 and 5, then finally formed β-sheet-rich structures which minimum wavelength at 217 nm on day 6 (Fig. 1C). For the spectra of Aβ42, the random-coil-like conformation on day 0 quickly moved to β-sheet-rich structure after day 1 (Fig. 1H), this result is consistent with the result of ThT assay showing Aβ42 forms fibrils much faster than Aβ40. When Zn^2+^ was mixed with Aβ40 and Aβ42, the ellipticity was significantly reduced on and after day 0 (Fig. 1D,I). The result showed that Zn^2+^ affects the secondary structure of Aβ rapidly and the aggregates contain different secondary structures than Aβ alone. Previously, this reduction was also found in the presence of Al^3+^ and other metal ions in our previous findings with a different Aβ preparation method^33^. Interestingly, we found Zn^2+^ affects Aβ40 much more than Aβ42. The CD spectra of Aβ40 with Zn^2+^ did not show much secondary structure from day 1 to even after 6 days of incubation, whereas Aβ40 without Zn^2+^ formed β-sheet fibrils readily. In the presence of Zn^2+^ with Aβ42, the random-coil-like structure was also reduced at day 0 and transformed to mixed spectra after 1 day (Fig. 1I). On day 2, there is no obvious β-stand formation unlike the Aβ42 alone (Fig. 1H). The single wavelength changes of Aβ40 (Fig. 1E) and Aβ42 (Fig. 1J) at 200 and 220 nm are plotted against time after the addition of Zn^2+^. The results showed that Zn^2+^-induced Aβ changes were rapid and complete in minutes.

### ZnAβ40 and ZnAβ42 form oligomers that are less heterogeneous and possess higher hydrophobic-exposed surfaces than ADDLs

**Figure 2:**
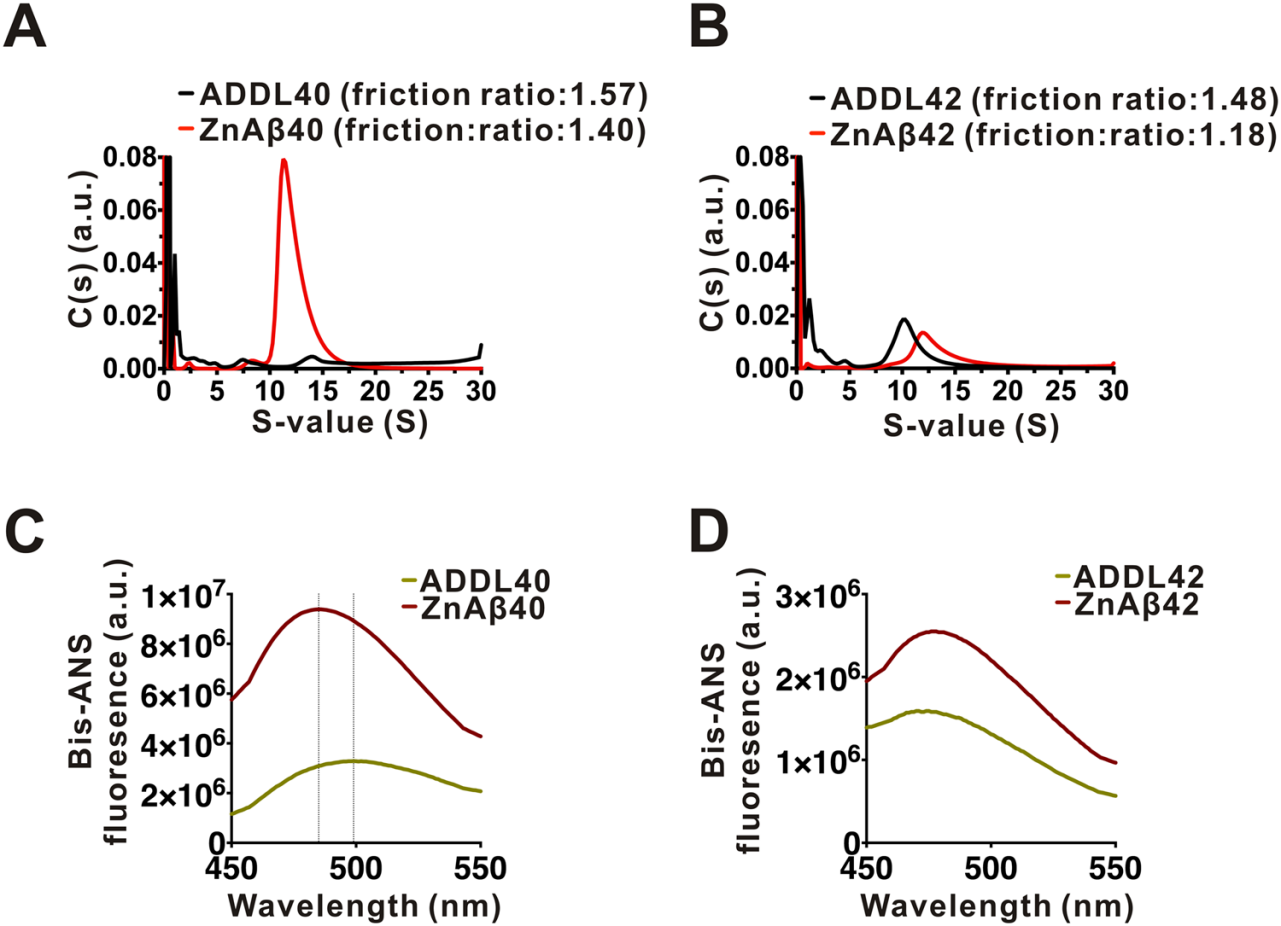
ZnAβ40 or ZnAβ42 assemble into oligomers and contain more exposed hydrophobic surfaces than ADDLs. (**A**,**B**) SV in AUC of ZnAβ or ADDLs consist of Aβ40 or Aβ42, respectively. The data were analyzed by a continuous c(s) distribution model and the sedimentation coefficients were calculated and shown. (**C**,**D**) Bis-ANS spectra of ZnAβ and ADDL. ZnAβ40, ZnAβ42, ADDL-Aβ40, and ADDL-Aβ42 at 100 µM were prepared. Before the measurement, all Aβ solution were diluted to 50 µM in 10 mM Tris-HCl, pH 7.4, then, mixed with 0.5 µM Bis-ANS dye. Bis-ANS fluorescence spectra were collected. The spectra were subtracted from the respective buffer control.

To estimate the size of ZnAβ oligomers, we analyzed ZnAβ40 and ZnAβ42 oligomers and ADDLs composed of Aβ40 or Aβ42 by sedimentation velocity (SV) in analytical ultracentrifugation (AUC). ADDL is a type of Aβ oligomers commonly reported in the literature that are cytotoxic^25,47,48^. ADDL was prepared as described in method according to previous literatures^25,48,49^. The SV result showed that the predominant sedimentation coefficient distribution of ZnAβ40 or ZnAβ42 were similar. They ranged from 10 to 17.5 S corresponding to molecular weight ranging from 152 to 214 kDa (~33 to 49 mer). The peak maxima were situated at 11.3 and 11.9 S, respectively, where the peaks contained ~63% and ~46.2% of total Aβ (Fig. 2A,B). In the condition to form ADDLs by Aβ40, the major distribution were broadly distributed which indicates no distinct oligomer formed in this condition. This result is consistent with the previously report that no Aβ40 oligomer was observed under AFM in ADDL preparation^25^. In ADDL made by Aβ42, there is a major peak situated at ~10.27 S which corresponds to molecular weight of 159 kDa (~35 mer), containing ~26.09% of total peptide. There are some other assemblies presented under 5 S which corresponds to molecular weight less than ~95 kDa (~21 mer). The friction ratio of ZnAβ40 and ZnAβ42 are 1.4 and 1.2, whereas, ADDL40 and ADDL42 are 1.57 and 1.48, respectively. Since the perfect sphere has a fraction ratio of 1.2, the result indicates that ZnAβ complexes are more spherical. Our AUC data demonstrated that ZnAβ40 and ZnAβ42 form oligomers that are less heterogeneous and more spherical than ADDLs.

Besides AUC analysis, we employed Bis-ANS fluorescence to monitor the hydrophobic clusters exposed on Aβ surface^18,50,51^. The experiments were performed with 50 µM ZnAβ oligomers or ADDLs. The samples were mixed with Bis-ANS readily within 1 min and subjected to fluorescence measurement. The fluorescence spectra of Aβ in the presence of Zn^2+^ ion was shown to be ~2.8 and ~1.6 fold higher than that of ADDLs Aβ40 or Aβ42, respectively (Fig. 2C,D). This enhancement indicated higher level of hydrophobic surface exposed on the protein surface of ZnAβ oligomers. Besides increasing of fluorescence intensity, the spectra of ZnAβ40 oligomers also showed blue-shift of maximal wavelength from 499 nm to 485 nm, but the shift in the ZnAβ42 is not that obvious, indicating that the presence of Zn^2+^ altered the Bis-ANS binding environment.

### Low immunoreactivity of ZnAβ oligomers reveal distinct oligomeric structure

To further elucidate the effect of Zn^2+^ on conformation changes of Aβ, we collected time-course Aβ aggregates for dot blotting analysis to monitor epitope changes (Fig. S5). Aβ40 and Aβ42 samples at different time points of incubation were dotted on the nitrocellulose membrane with several replicates and probed by several Aβ antibodies including conformational specific antibodies A11 and OC and sequence specific antibodies like 6E10, 4G8, A3356, and C-terminal antibody. A11 antibody was used to detect common prefibrillar oligomers^52^, whereas OC antibody recognizes fibrils and fibrillar oligomers, which are structurally and immunologically related to fibrils^53,54^. The sequence specific antibodies used were 6E10 antibody recognizing N-terminal Aβ residues 1–16, 4G8 recognizing Aβ residues 17–24, A3356 recognizing the middle terminus of Aβ residues 22–35, anti-C-terminal of 42 recognizing the last 6 residues of Aβ42. The peptide loading control is detected by Direct Blue staining. From the different time points collected during Aβ40 and Aβ42 aggregation incubated with or without Zn^2+^ (Fig. S3A and S3B), we found A11 signals of Aβ40 increased after day 1, and that of ZnAβ40 increased after day 5. Both of the signal saturated after 6 days. In the OC blot, we found the signals of Aβ40 potently increased after day 1, but ZnAβ40 showed almost no OC signal in comparison to Aβ40 alone. Similarly, A11 and OC signals were both much weaker in the ZnAβ42 blot compared with Aβ42 alone. These evidences suggest that ZnAβ oligomers revealed distinct immunological status compared with Aβ aggregates in the absence of Zn^2+^. The data are consistent with the results from ThT assay and TEM imaging demonstrating no or little fibrillary structure is formed in ZnAβ. Surprisingly, we found that even sequence-dependent antibodies such as 6E10 or 4G8 showed weaker reactivity to recognize ZnAβ40 and ZnAβ42 while we have equal Aβ loading on the blot as shown by Direct Blue staining. Furthermore, we compared the epitopes in ZnAβ oligomers and ADDLs by the different antibodies. We dotted ZnAβ incubated for 1 and 7 days with ADDLs that were prepared in media for 1 day as described in the method. We found that both ZnAβ40 and ZnAβ42 incubated for 1 or 7 days showed weaker immunoreactivity compared with the corresponding ADDLs (Fig. S5C and S5D). This result further demonstrated that ZnAβ oligomers expose distinct epitopes and maintain a quite different conformation than ADDLs and the intermediates in Aβ aggregation without Zn^2+^.

### ZnAβ40 oligomers are structurally more disordered than Aβ40 fibrils

**Figure 3:**
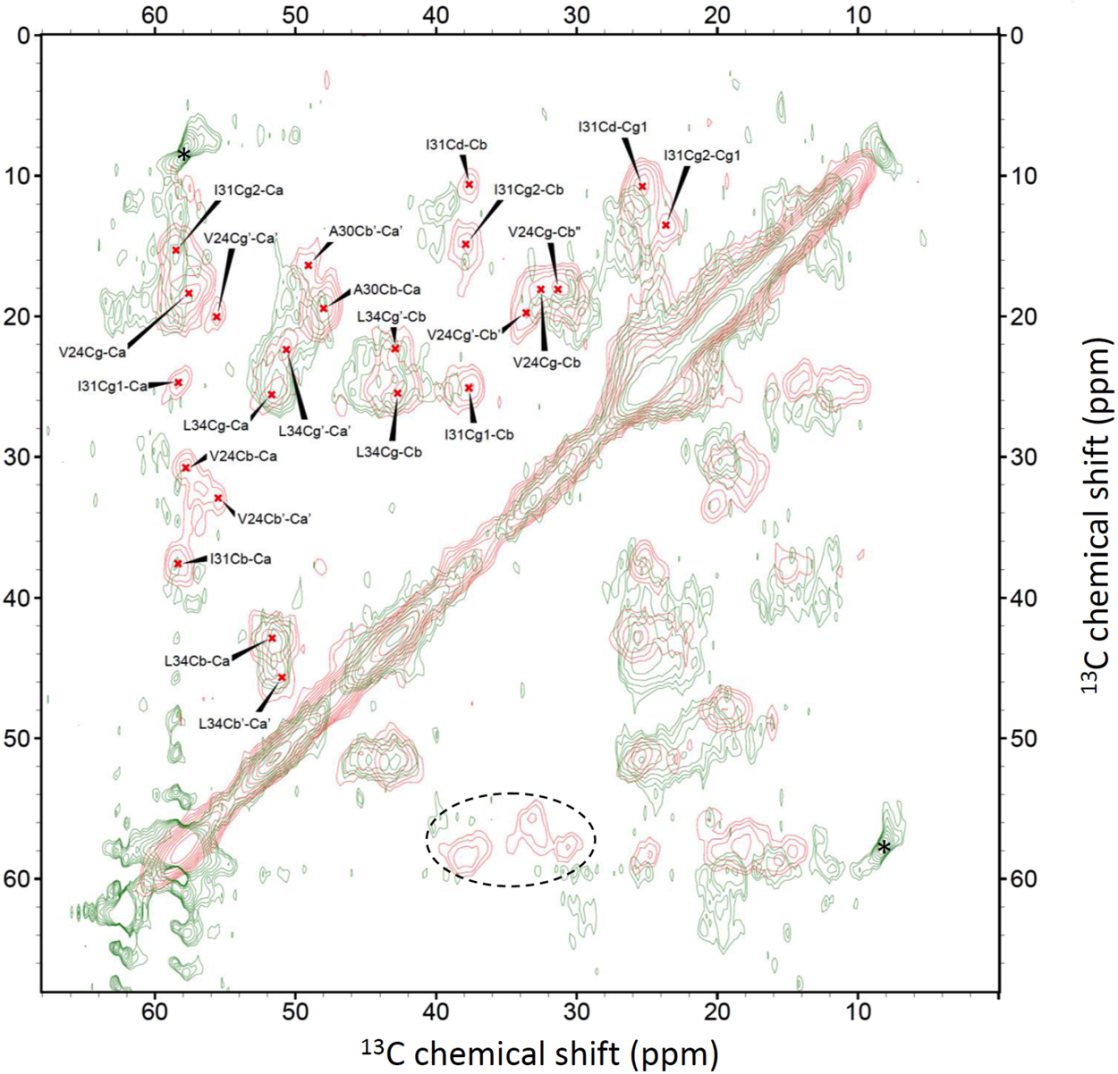
Overlaid ^13^C homonuclear correlation spectra of ZnAβ40 (green) and Aβ40 fibrils (red). The Aβ40 peptides were ^13^C enriched at V24, A30, I31, G33, and L34. Spectral assignments were given for the spectrum of the Aβ40 fibrils. The dashed elliptical circle highlighted the region of the C^α^/C^β^ cross peaks of V24 and I31 expected for ZnAβ40. The peaks denoted by asterisks were spinning sidebands.

It is well known that the chemical shifts of CO, C^α^, and C^β^ are sensitive to the backbone conformations of polypeptides^55–57^. To understand the molecular structure of ZnAβ, we first examined the ^13^C uniformly labeled ZnAβ40 by solid-state homonuclear ^13^C correlation spectrum. In contrast to the highly resolved NMR spectra of Aβ40 fibrils^58^, the resolution of the spectrum acquired for our ZnAβ40 sample was very poor (Fig. S6). It implied that ZnAβ40 still exhibited a significant structural heterogeneity at the molecular level. This finding suggested that the formation of ZnAβ40 aggregates is a kinetically controlled event. To tackle the resolution problems, we prepared a selectively labeled ZnAβ40 sample in which only the residues of V24, A30, I31, G33, and L34 were ^13^C labeled. The corresponding NMR spectrum is shown in Fig. 3. The assignments of the ZnAβ40 spectrum are given in the Supporting Information (Fig. S7). Accordingly, most of the cross peaks of V24 were significantly attenuated and only the C^β^/C^γ^ cross peak was barely observed. The C^α^/C^β^ cross peak of A30 was discernible but that of I31 was not observed. Nonetheless, the cross peaks of the side chain carbons of I31 remained observable. The CO/C^α^ cross peak of G33 were observed but its line width was relatively large, rendering an accurate determination of its chemical shifts difficult. The cross peaks of L34 were the most intense in the ZnAβ40 spectrum and there were at least two sets of resonances. For comparison, the labeled peptides were also incubated under quiescence conditions to form Aβ40 fibrils. Figure 3 showed the overlay ^13^C NMR spectrum of ZnAβ40 and Aβ40 fibrils. The chemical shifts and line widths of the backbone carbons of L34 were rather similar between ZnAβ40 and Aβ40 fibrils. On the other hand, the chemical shifts of ZnAβ40 revealed that the residue A30 is largely in the β-sheet conformation, but to a lower extent than Aβ40 fibrils. In addition, the line widths of the A30 signals of ZnAβ40 were larger than those of Aβ40.

### Removal of Zn^2+^ restores amyloid fibril formation with a faster kinetics and ZnAβ is unable to serve as fibrillization seeds

**Figure 4:**
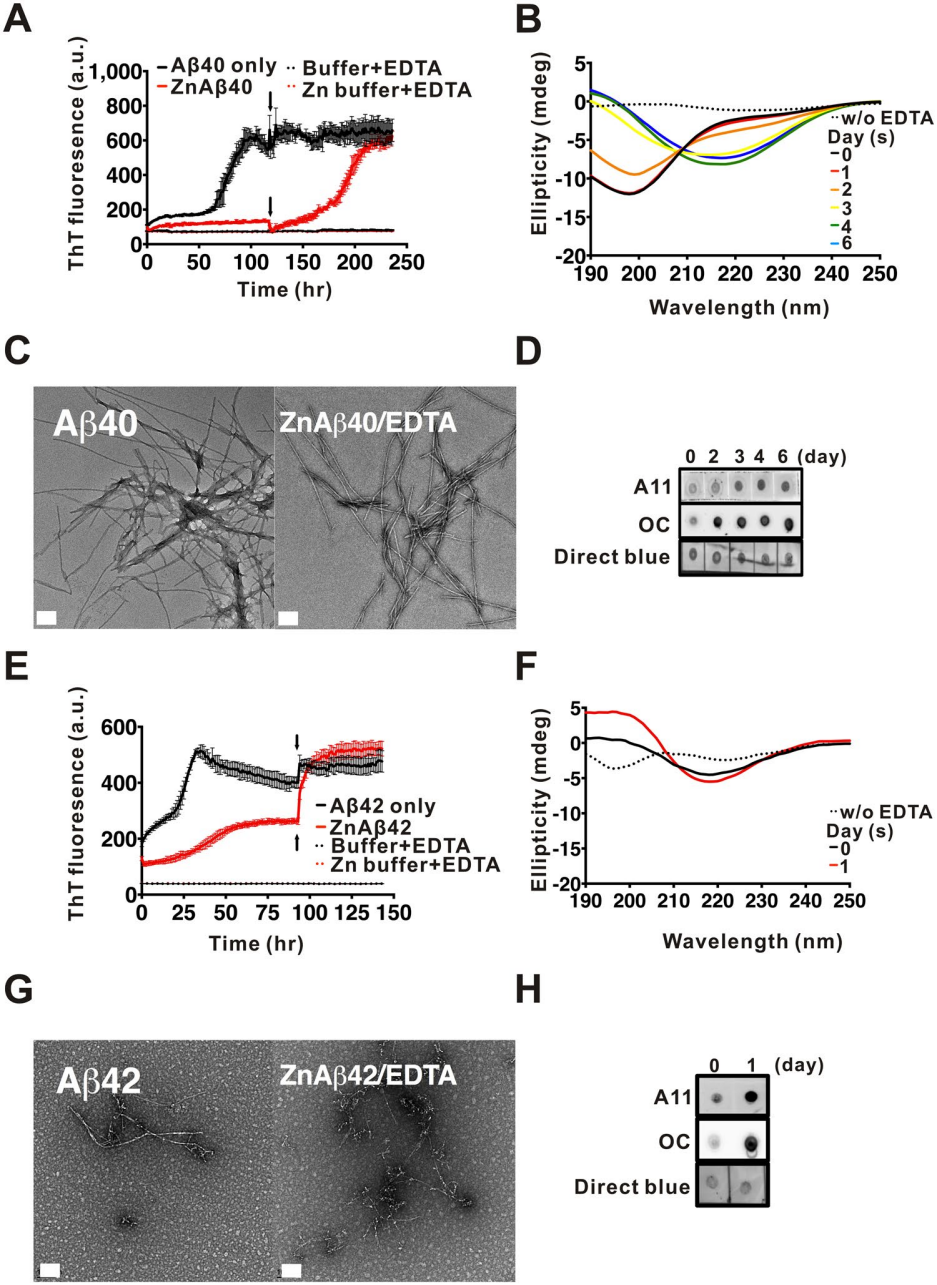
ZnAβ40 oligomer can be restored rapidly back to fibrillization pathway by EDTA. (**A**,**E**) Aβ and ZnAβ40 (**A**) or ZnAβ42 (**E**) aggregation monitored by ThT assay. Three folds of EDTA was added when Aβ fibrillization reached a plateau. The time of addition is indicated by arrows. (**B**,**F**) Time-course conformation changes of ZnAβ40 or ZnAβ42 after EDTA treatment measured by far-UV CD. (**C**,**D** and **G**,**H**) TEM images (**C** and **G**) and dot blotting (**D**,**H**) of the time course samples in the aggregation. Scale bars in TEM images are 100 nm. ZnAβ was first prepared and incubated for 1 day for ZnAβ oligomer formation prior to EDTA treatment in the CD and dot blotting experiments. Dot blotted membrane was probed with A11 and OC antibody and stained by Direct blue as a dotting control.

Since the presence of Zn^2+^ promoted Aβ40 or Aβ42 forming stable oligomers, we wonder whether metal ion chelator, such as EDTA, can disrupt the interaction of Zn^2+^ ions and Aβ. Here, we added three-fold molar concentration of EDTA into the ZnAβ or Aβ solution after plateau phase of ThT intensity at the time point indicated by arrows and monitored the conformation and morphology by far-UV CD and TEM image (Fig. 4). Aβ40 or Aβ42 in the absence or presence of Zn^2+^ were incubated and monitored by ThT assay (Fig. 4A,E). When the ThT intensity of Aβ alone reached a plateau phase, EDTA were added to both solution. We found that upon adding EDTA to trap Zn^2+^ ions, ZnAβ40 showed a rapid restoration to form amyloid fibrils with a short lag phase (<~40 hr), whereas ThT intensity of Aβ40 alone was not changed after EDTA addition (Fig. 4A). We also monitored the secondary structural changes by time-course far-UV CD spectra (Fig. 4B). Interestingly, the result showed that removal of Zn^2+^ ions immediately promoted ZnAβ40 to again show random-coil-like structure (labeled as day 0) similar to the Aβ40 monomer solution without Zn^2+^. The initial spectra of ZnAβ before addition of EDTA was flat (labeled as dash line). After EDTA treatment, Aβ gradually transformed to β-sheet-rich structure after 4 days of incubation. This transition is similar to conformational transition (6 days) in Aβ alone but faster. We monitored the morphology of the end-point products by TEM (Fig. 4C) and found ZnAβ40 after EDTA treatment has formed mature fibrils similar to the control Aβ without Zn^2+^. Dot blotting by A11 and OC antibody of the time course ZnAβ40 samples after EDTA treatment also demonstrated that trapping of Zn^2+^ by EDTA results in restoration of Aβ40 to fibrillization pathway (Fig. 4D). In the case of Aβ42, ThT assay showed that EDTA treatment also promoted ZnAβ42 to immediate formation of amyloid fibrils without a lag phase (Fig. 4E). However, in far-UV CD analysis, we found the spectra removal of Zn^2+^ ions by EDTA still showed the β-strand content which may be due to unable to monitor the rapid aggregation of Aβ42. Dot blotting and TEM further confirmed the restoration of ZnAβ42 into amyloid fibrils and restoration of the immunoreactivity (Fig. 4G,H).

**Figure 5:**
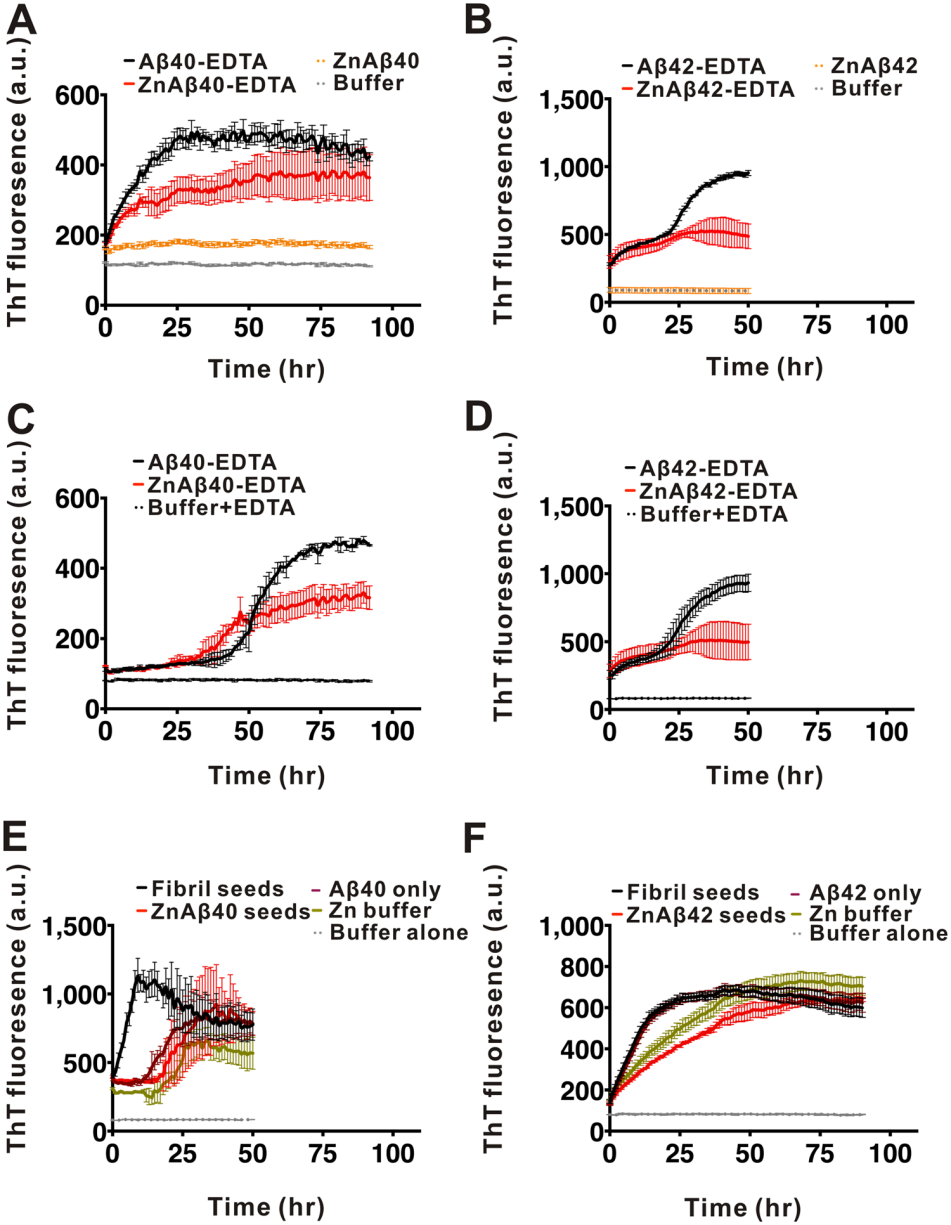
EDTA-treated ZnAβ40 showed faster fibrillization rate and ZnAβ oligomers are unable to seed Aβ. (**A**,**B**) Fibrillization kinetics of (**A**) EDTA-treated ZnAβ40 or Aβ40 and (**B**) EDTA-treated ZnAβ42 or Aβ42 after seeding. Five % sonicated preformed fibril seeds were added into EDTA-treated ZnAβ and Aβ solution. Fibrillization kinetic was measured by ThT fluorescence. ZnAβ alone and buffer containing 5% fibril seeds were also monitored. (**C**,**D**) Fibrillization kinetics of Aβ40 (**C**) and Aβ42 (**D**) with or without Zn^2+^ after EDTA treatment. ZnAβ was first prepared and incubate for 1 day for oligomerization, then added EDTA to remove Zn^2+^ ion. Aβ alone solution was also treated with EDTA for comparison. Fibrillization kinetics was measured by ThT fluorescence. (**E**,**F**) Seeding experiment were performed by adding Aβ fibrils or ZnAβ40 (**E**) or ZnAβ42 (**F**) as seeds. The respective buffer were added to Aβ solution as negative control.

Next, we would like to examine the property of Aβ-EDTA and ZnAβ-EDTA being seeded. We performed seeding experiment by addition of fibril seeds generated from preformed Aβ fibrils to soluble Aβ-EDTA and ZnAβ-EDTA solution and monitored the fibrillization kinetics by ThT fluorescence (Fig. 5A,B). The preformed Aβ40 or Aβ42 fibril seeds were sonicated and added in 5% to respective soluble Aβ solution. The result showed that all Aβ-EDTA and ZnAβ-EDTA can be seeded by fibril seeds showing accelerated fibrillization. There is no difference in initial kinetic rate of fibrillization, but the plateau phase of ThT intensity of ZnAβ-EDTA was markedly lower than Aβ-EDTA. This may indicate a distinct ThT binding environment or fibril structure formed from ZnAβ-EDTA fibrillization. Furthermore, we directly compared the fibrillization kinetics of Aβ and Zn-Aβ both treated with EDTA (Aβ-EDTA and ZnAβ-EDTA) by ThT assay (Fig. 5C,D). The result showed that the aggregation kinetics of ZnAβ40-EDTA was notably faster than Aβ40-EDTA with a shorter lag phase (ZnAβ40-EDTA, lag time = 33 hr; Aβ40-EDTA, lag time = 42 hr) (Fig. 5C), whereas, ZnAβ42-EDTA still showed similar kinetic profile as Aβ42-EDTA alone (Fig. 5D). The plateau phase of ThT intensity of both ZnAβ40-EDTA and ZnAβ42-EDTA were markedly lower than the respective Aβ-EDTA. The result is consistent with observing faster aggregation after Zn removal in aforementioned experiments (Fig. 4A,B). It suggests ZnAβ oligomer may facilitate formation of an Aβ conformation that is less heterogeneous and can facilitate the Aβ fibrillization after removal of Zn^2+^. However, the effect is not easily seen in Aβ42 that may be due to fast aggregation of Aβ42 hindering the observation of the difference.

We further seeded preformed Aβ fibrils and ZnAβ oligomers to Aβ solution to examine the seeding property of ZnAβ in comparison to Aβ fibrils (Fig. 5E,F). Aβ40 and Aβ42 fibril seeds or ZnAβ40 and ZnAβ42 seeds were add 5% to the Aβ solution. The corresponding buffers were also added separately as negative controls. We found that Aβ fibril seeds can greatly accelerate fibrillization as expected. In Aβ40, the ThT signal of Aβ with fibril seeds elongated immediately without a lag phase compared to Aβ without fibril seeds. Whereas, Aβ with ZnAβ40 seeds did not show acceleration but adopted a similar aggregation characteristic of Aβ without seeds (buffer added). Similarly, in Aβ42, Aβ42 with Aβ42 fibril seeds adopted a faster elongation rate in comparison to the Aβ42 without seeds. ZnAβ42 oligomer did not seed Aβ42 solution. The results demonstrate ZnAβ cannot serve as seeds to accelerate fibrillization. Overall, the seeding experiments showed consistent results to indicate off-pathway property of ZnAβ.

### ZnAβ oligomers possess higher cytotoxicity than ADDLs

**Figure 6:**
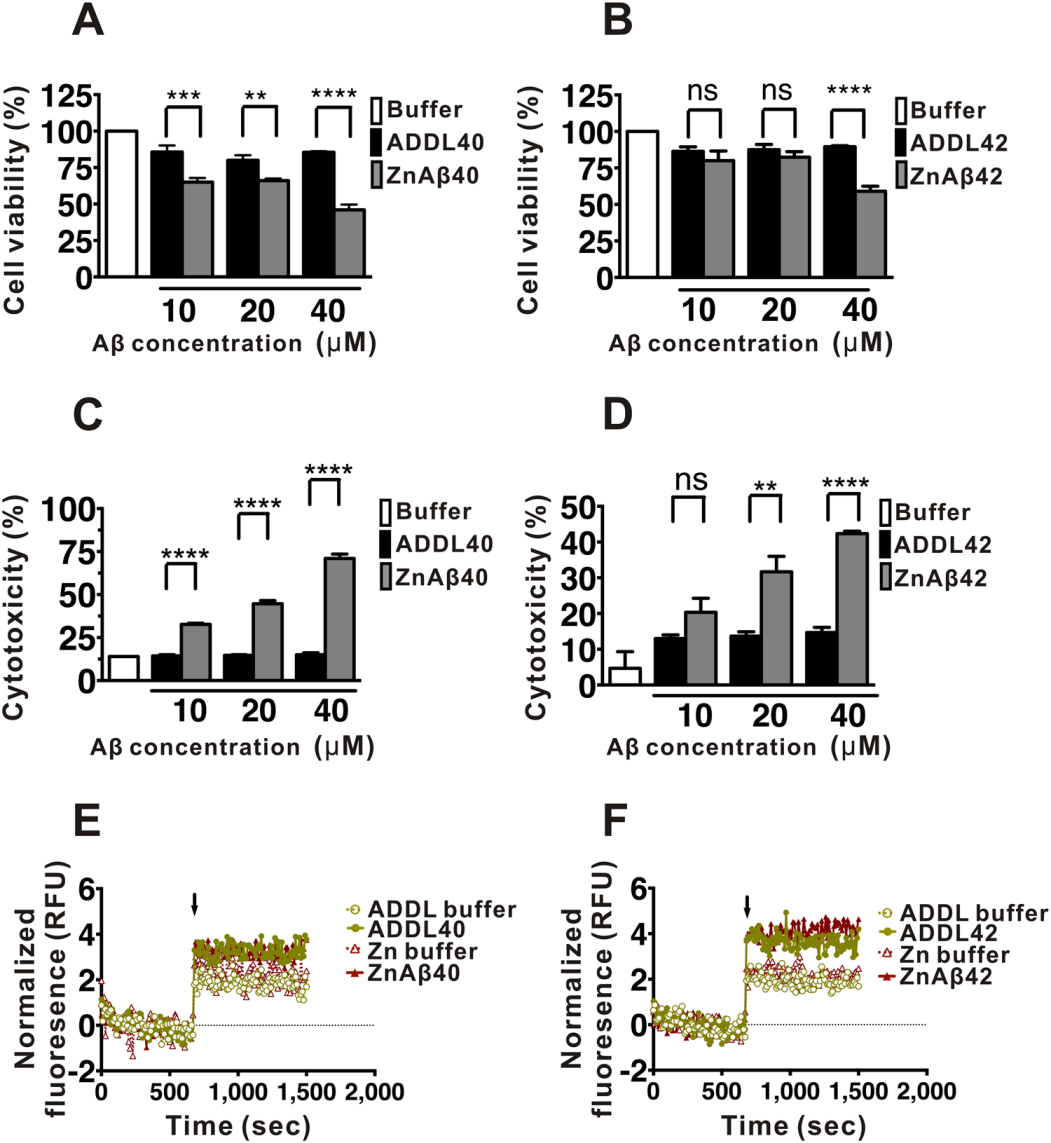
ZnAβ oligomers show stronger reduction to cell viability and are more cytotoxic than ADDLs, but they induce similar Ca^2+^ influx. (**A**,**B**) Cell viability was examined by MTT assay for ZnAβ and ADDLs treatment. ZnAβ and ADDLs in different concentrations were treated to neuroblastoma cells. (**C**,**D**) Cytotoxicity assay by LDH for ZnAβ and ADDLs treatment. The percentage of LDH release was calculated by the slope of fluorescence intensity compared with maximum LDH release control. Triton-X 100 treated cell was served as 100% cytotoxicity. (**E**,**F**) Intracellular Ca^2+^ was monitored by Fluo-3 AM dye. ZnAβ samples were filtered prior to experiment. Before treatment, cell was first incubated with dye and measured for basal level of fluorescence intensity for 10 mins. Aβ was added as indicated arrow. The statistical analysis was performed by one-way ANOVA and Turkey Post Hoc Test, where p < 0.05 (*), <0.01 (**), <0.001 (***), <0.0001 (****). The averaged values were shown with standard divisions.

Aβ oligomers have been proposed to be the major cause contributed to neurotoxicity in the Aβ assembly. Here, to understand whether ZnAβ oligomers show difference in the biological effects compared with ADDLs, we examined the cell viability and cytotoxicity contributed from ZnAβ oligomers and ADDLs. Since high Zn^2+^ ion concentration induces cell death^59,60^, we exchanged excess Zn^2+^ in the preparation with 10 mM Tris-HCl buffer, pH 7.4 by MWCO 3 KDa centrifugal filter and re-quantify concentration prior to further test. The remaining Zn concentration after exchanged was 2 µM (data not shown). The cell viability and cytotoxicity of the oligomers were measured in human neuroblastoma BE(2)-C cells by MTT and LDH assays, respectively. After 24 hr of incubation with ZnAβ or ADDL at concentration ranging from 10 to 40 µM, both ZnAβ or ADDL oligomers showed reduction of cell viability. In general, ZnAβ was able to induce dose-dependent cytotoxicity. At 40 µM, ZnAβ40 and ZnAβ42 reduced 54% and 41% of cell survival respectively, whereas, ADDL40 and ADDL42 reduced 15% and 11% of cell survival, respectively (Fig. 6A,B). Besides MTT assay for cell viability, we also used LDH assay to interpret whether these oligomers increased cytotoxicity. The LDH assay showed both ZnAβ significantly increased the percentage of cytotoxicity in a dose-dependent manner. At 40 µM of ZnAβ40 and ZnAβ42 increased cytotoxicity to 71% and 42%, respectively, whereas ADDL40 and ADDL42 increased 15% and 14% of cytotoxicity, respectively (Fig. 6C,D). Besides cell viability and cytotoxicity assays, Ca^2+^ influx is one of the Aβ oligomer-induced adverse effect that promotes apoptosis^61^. Here, we employed Ca^2+^ binding dye, Fluo-3 AM, to detect Ca^2+^ influx. Fluo-3 AM dye is cell permeable and emits fluorescence by intracellular esterase activity. The cells were subjected to ZnAβ or ADDLs of Aβ40 or Aβ42 at 40 µM and the fluorescence signals were measured after sample addition (Fig. 6E,F). From the result, we found both ZnAβ40 and ZnAβ42 were able to increase Ca^2+^ influx levels as indicated by the fluorescence intensity and the level are similar to ADDLs. Overall, the result demonstrated that ZnAβ40 and ZnAβ42 are more toxic than ADDLs but they induce similar effect in Ca^2+^ influx.

### ZnAβ oligomers inhibit hippocampal LTP and increase microglial activation in wild-type mice

**Figure 7:**
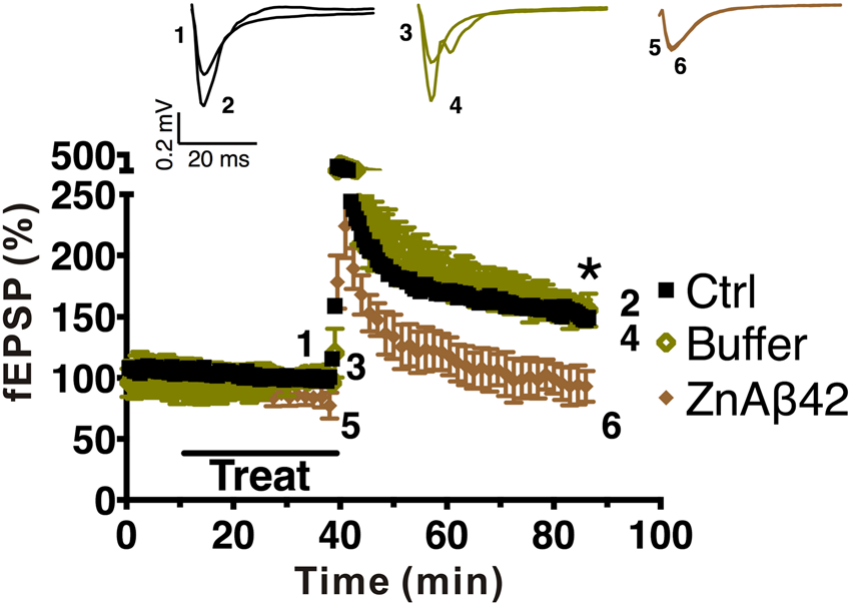
ZnAβ42 oligomers inhibit LTP in wild-type mice hippocampal slice. Filed EPSPs (fEPSP) were first measure for stable base line, then treated with filtered ZnAβ42 or buffer control for 30 min (ctrl, n = 5, buffer, n = 3, ZnAβ42, n = 3). LTP was induced by theta burst stimulation given at baseline intensity after treatment. Insets show example fEPSP traces before and post theta-burst stimulation (stimulus artifact was removed for clarity). The statistical analysis were performed by two-way ANOVA and Turkey Post Hoc Test (*p < 0.05).

Previous studies have demonstrated toxic effect of ADDLs on synaptic plasticity and long-term potentiation (LTP). LTP is a good paradigm to investigate synaptic plasticity and a model of learning and memory^62–64^. Therefore, to further assess the effect of ZnAβ oligomer on synaptic plasticity, we treated 500 nM ZnAβ42 oligomer for 30 mins on the hippocampal slice from wild-type C57BL/6JNarl mice since Aβ42 is considered the more toxic Aβ species. The ZnAβ42 oligomers were filtered before the experiments to remove excessive Zn ions. In LTP results, we found significant LTP impairment generated by ZnAβ42 oligomers compared to the buffer treated and control one. Hippocampal slices treated with ZnAβ42 showed LTP-impairment for 47 mins after tetanic stimulation (mean amplitude 89.44 ± 13.91%), whereas, control and filtered Zn buffer-treated hippocampal slice induced LTP within the amplitude of 147.5 ± 16.27% and 152 ± 9.29% (Fig. 7). This result indicated the effect of ZnAβ oligomers to interfere synaptic plasticity.

**Figure 8:**
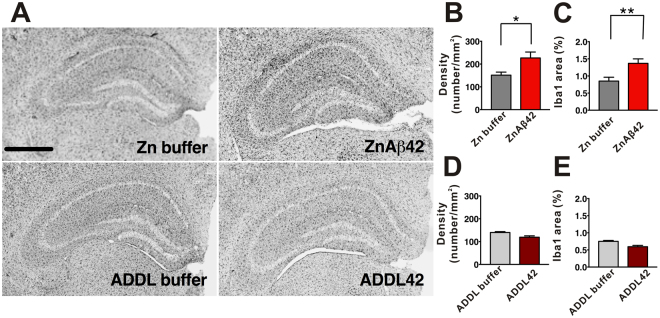
Effect of ZnAβ42 oligomer on hippocampal microglia activation in the wild-type mice. (**A**) The micrograph represents the Iba1^+^ positive microglia in the hippocampus of the mice after ZnAβ42 or ADDLs injection. The quantitative results of Iba1^+^ microglia are represented as density (**B**,**D**) and area (%) (**C**,**E**) (ZnAβ42, n = 19, Zn buffer, n = 23, ADDL42, n = 24, ADDL buffer, n = 17, and n values are from the brain slices of 3 mice of each group). The statistical analysis was performed by unpaired Student’s t test where p < 0.05 (*), <0.01 (**). Scale bar = 800 µm.

To investigate whether ZnAβ oligomers prepared *in vitro* showed neurotoxicity in the mice brain, we injected 40 µM ZnAβ or ADDL42 oligomers into dorsal hippocampus of the WT mice brain as an acute Aβ-injected model. After 14 days, the mice were sacrificed and the brain slices were subjected to immunohistochemistry stained with Iba1 antibody for microgliosis and GFAP antibody for astrogliosis. The representative Iba1 (Fig. 8A) and GFAP staining (Fig. S8) in the hippocampus were shown and the quantified results were calculated. The results showed that the mice received injection of ZnAβ oligomers have ~20% increase of microglial density and ~30% increase of microglial area in the hippocampus region compared with the respective buffer injected group (Fig. 8B,C). Relatively, ADDL-injected group did not show obvious microglia activation compared with its buffer injected group (Fig. 8D,E). The extent of astrogliosis was also measured by density and area stained by GFAP antibody, however it seems no increase of density and area in the mice received ZnAβ or ADDL42 compared with its respective buffer injected groups (Fig. S8B to S8E).

## Discussion

Zn^2+^ homeostasis and synaptic Zn^2+^ levels play crucial role in the AD pathology^65,66^. In the glutamatergic synapses, storage of Zn^2+^ is concentrated by the ZnT3 transporter as a pool achieved up to ~300 µM^31^. In the physiological condition, extracellular Zn^2+^ concentration is quite low (<1 µM), and may be released up to exceed 100 µM from the synaptic cleft at the peak of neuron activity^67^. Zinc ion has been reported to play both neuroprotective and neurotoxic roles. In previous literature, the neurotoxicity of Aβ reduces in the presence of Zn^2+^ at sub-stoichiometric concentration^42,68^, whereas, the neurotoxicity of Aβ increases *in vitro* and *in vivo* at stoichiometric concentration of Zn^2+^ ^68,69^ with some exceptions in different experimental conditions^70^. Besides, knockout of ZnT3 transporter in AD mice have marked reduction of amyloid plaque load^65^. In the present study, we focused on characterizing biological and structural concepts of Zn^2+^-induced Aβ oligomerization. We first showed the influence of equal molar concentration of Zn^2+^ on Aβ40 or Aβ42 that significantly altered the structure and pathway of Aβ fibrillization resulting in oligomerization as evidenced by their low ThT and far-UV CD signals, larger hydrophobic exposed surfaces by Bis-ANS fluorescence, oligomer assembly by AUC, oligomeric morphology in TEM, and altered immunoreactivity. The effect is limited in the initial stage of aggregation but not to the preformed fibrils. Interestingly, removal of Zn by EDTA not only rapidly restored Aβ fibrillization but also adopted a faster kinetics. We also demonstrated the ZnAβ oligomers potently inhibited hippocampal LTP and initiate microglia activation in acute Aβ-injected model. Our result demonstrated that Zn^2+^ plays a key role in the conformation and oligomerization of Aβ in which the species is highly toxic.

On the basis of the ^13^C solid-state NMR spectrum of uniformly labeled ZnAβ40, we found that the overall structure of ZnAβ40 was highly disordered. Nonetheless, the spectrum acquired for the selectively labeled ZnAβ40 revealed some site-specific structural insights for ZnAβ40. With reference to the published backbone conformation of Aβ40 fibrils^71^, the fold of the peptides adopts a motif of β-sheet/turn/β-sheet. The residue V24 is in the turn region, whereas A30 to L34 are in the second β-sheet region. Our chemical shift data of ZnAβ40 suggested that the residue V24 was very structurally disordered and/or the residue might undergo considerable motional dynamics. Interestingly, we found that the residues A30 and L34 of ZnAβ40 largely adopted a β-sheet conformation, although we did not prepare a series of selectively labeled samples to map out the sequence location of the β-sheet structure. Recently, it has been shown by solid-state NMR that Zn^2+^ ions would modulate the structure of the aggregates of ZnAβ40 and ZnAβ42^16,72^. In comparison, our chemical shift data at A30 and L34 are consistent with the data reported by Madhu and co-workers for ZnAβ40^16^, whereas the signal line widths of our data were considerably larger (Fig. 3). Because our ZnAβ40 sample was prepared with a relatively short incubation time, the sample was structurally more heterogeneous than the corresponding fibrillar aggregates, as revealed in the solid-state NMR spectra. At the present stage, it is not clear whether the elevated cytotoxicity of ZnAβ40 is due to the structural flexibility of ZnAβ40, or a small subset of the structural isoform of ZnAβ40 is particularly cytotoxic. In view of the poor spectral resolution for ZnAβ40, we did not attempt to measure the NMR spectrum for ZnAβ42.

Aβ oligomer among Aβ aggregates is suggested as the actual toxic species to impair synapse function in AD pathology^8,9,62^. The aggregation pathway initiated with misfolded monomer can be oligomerized to on-pathway or off-pathway oligomers. For example, fibrillary oligomers with OC antibody-positive signal are suggested as on-pathway oligomer within the ability to directly serve as fibril nuclei or seeds to elongate by adding free monomers to their ends^11^. However, monomer might aggregate into prefibrillar oligomer, A11-positive, OC-negative signals, as off-pathway oligomer, which cannot directly elongate into form mature fibrils without first form protofibrils and further conformation changes^52,53^. EGCG interaction with Aβ promotes the formation of off-pathway non-toxic Aβ oligomers^73^. Our immunodotting results showed ZnAβ oligomers possess lower affinity with most anti-Aβ antibodies including A11 and OC. The result suggest novel properties of ZnAβ oligomers. As shown in our AUC result for the first time that ZnAβ oligomers are less heterogeneous than ADDLs, the reported culprit in AD that are often used in many biological papers^74–76^. From EDTA experiments, we found EDTA recovered ZnAβ fibrillization by restoring ZnAβ40 to random coil-like structure and followed by a faster fibrillization kinetics as evidenced by ThT assay, far-UV CD, and dot blotting. The results suggest ZnAβ oligomers are off-pathway species that are trapped by Zn ion. Once Zn is removed, Aβ rapidly restores its properties to undergo fibrillization. Although we only capture ZnAβ40, but not ZnAβ42, returned to random coil structure after Zn removal, the faster kinetics of Aβ42 fibrillization may be the reason hinder the observation. We hypothesized that Zn trapped Aβ oligomer concentrated local Aβ that facilitates the faster fibrillization after Zn removal.

Although both ZnAβs and ADDLs form oligomers, they have substantial amount of differences. Firstly, ZnAβ oligomers expose larger hydrophobic surface area and show lower immunoreactivity against Aβ antibodies than ADDLs. Besides, ZnAβ oligomers are stable oligomers even incubation at room temperature, whereas, only Aβ42 ADDLs but not Aβ40 ADDLs form in a special condition at 4 °C^25^. Comparing to our data and previously literature^74^, our AUC result confirmed the narrower distribution of ZnAβ oligomers. The higher toxicity of ZnAβ compared to ADDL also strengthen the pathogenic role of ZnAβ in AD. Zn and Aβ coordination has been proposed for Zn^2+^ using the three histidines and the N-terminus^39,40^. Inter-molecular Aβ-Zn coordination has also been reported via histidine-bridges^77–80^. Although N-terminal Aβ is not considered in the current oligomer and fibril models, manipulation of N-terminal Aβ indeed affected the Aβ aggregation and induced toxicity. For example, genetic variation in alanine 2 in Aβ sequence, or alanine 673 in APP, were reported to have either protective^81^ or adverse effect^82,83^ on AD. N-terminal Aβ inhibitor^84^ or antibody against the N-terminal region^85^ have been shown to have beneficial effect.

Synaptic activity has been reported as a critical role to control Aβ oligomers formation and targeting at synaptic terminals and Zn^2+^ was involved in this activity-dependent regulation^86^. Takeda *et al*. further supported the critical role of Zn^2+^ by demonstrating single Aβ injection stimulates Zn^2+^ influx and causes a short-term cognitive deficit^87^, while applying Zn^2+^ chelator can rescue the LTP impairment. They further proposed that interaction of Zn^2+^ and Aβ42 was essential for the rapid uptake of Zn^2+^ and Aβ42 into the rat dentate gyrus^88^. These results support the role of ZnAβ in AD pathogenesis. Clinical trials of passive immunization against Aβ aggregates has been a major therapeutic strategy. Recently, anti-Aβ antibody aducanumab has successfully shown cognitive improvement in mild AD patients in phase Ib clinical trial^89^. Future Aβ antibody development can also target ZnAβ oligomers. In conclusion, our aggregation and biological studies showed the aggregation mechanism of ZnAβ which facilitate the understanding the possible role of ZnAβ oligomers in the AD and provide a new insight to understand ZnAβ oligomer structure. This study may contribute to identify a potential therapeutic target in AD.

## Materials and Methods

### Aβ synthesis and preparation

Synthetic Aβ40 or Aβ42 peptides were acquired by solid phase peptide synthesis from the Genomics Research Center, Academia Sinica, Taiwan, as previously described. For selective-^13^C labeled Aβ40 were synthesized by the department of chemistry, National Taiwan University^90^. Aβ peptides were solubilized in 1,1,1,3,3,3-Hexafluoro-2-propanol (HFIP, Sigma-Aldrich, St. Louis, MO, USA) in 1 mg/mL to dissolve preformed aggregates. HFIP was lyophilized and then treated with 10% ammonium hydroxide (NH_4_OH) and lyophilized. Finally, lyophilized Aβ40 or Aβ42 were dissolved in 10 mM Tris-HCl buffer, pH 7.4. The Aβ solution was quantified by absorbance at 280 nm (ε = 1280 cm^−1^ M^−1^)^91^ to prepare working concentration. To prepare ADDL oligomers, lyophilized HFIP-treated Aβ40 or Aβ42 were dissolved in DMSO to 5 mM and sonicated for 5 mins. Then the dissolved Aβ was added to DMEM/F12 (GIBCO, Invitrogen). The concentration was quantified by absorbance at 280 nm to prepare 100 µM stock and incubated at 4 °C for 24 hr^25,49^.

### ThT Assay

Fifty µM Aβ solution was prepared in the presence of 5 µM ThT as previously described^92^. Briefly, the samples were incubated at 25 °C with agitating 60 sec each hr. Fluorescence of ThT was monitored at 485 nm by an ELISA microplate reader SpectraMax M5 (Molecular Devices, Sunnyvale, CA, USA) and excitation wavelength was at 442 nm. The experiments were repeated at least 3 times and averaged and presented as mean ± S.D.

### Dot blotting

Two µl of the each Aβ time-course samples were collected at a series of time points and dotted onto nitrocellulose membrane with several duplicates for probing by different antibodies. Membranes were probed with A11 (Invitrogen), OC (Millipore, Temecula, CA, USA) separately. Membrane was also staining with direct blue as a loading control to check equal loading of each dot.

### Direct blue staining

The staining solution of Direct blue 71 (Sigma-Aldrich, St. Louis, MO, USA) was prepared with 0.1% (w/v) in the double distilled water containing 40% ethanol, and 10% acetic acid. The nitrocellulose membrane with Aβ samples dotted was directly incubated in the staining solution and incubated for 10 min, then washed by double distilled water to acquire clear signals.

### Far-UV CD spectroscopy

Aβ samples were measured at a series of time points. Aβ solution was loaded into a circular quartz cuvette with 1 mm path length (110-QS, Hellma Analytics). Spectra were monitored from 250 to 190 nm by a J-815 CD spectropolarimeter (Jasco Inc., Easton, MD, USA). The data were accumulated 10 times of scans and averaged.

### TEM

The samples were dropped onto 400-mesh Formvar carbon-coated copper grids (EMS Electron Microscopy Sciences, Hatfield, PA, USA) and incubated for 5 mins and washed twice by double distilled water, then negatively staining was performed with 2% uranyl acetate for 2 mins and dried at room temperature overnight. Image were observed with a FEG-TEM, FEI Tecnai G2 TF20 Super TWIN transmission electron microscope with an accelerating voltage of 120 kV.

### Bis-ANS

Aβ samples were first mixed with 0.5 µM Bis-ANS solution and measured the fluorescence signal. The emission spectra of Bis-ANS were collected from 450 to 550 nm with excitation at 400 nm. Fluorescence emission spectra were obtained using a FluoroMax-3 spectrofluorometer (Horiba Jobin Yvon). The background from buffer control was subtracted.

### AUC

SV of AUC experiments were performed on a Beckman Optima XL-I analytical ultracetrifugation (Beckman Coulter, USA). ADDLs at 100 µM in DMEM/F12 or ZnAβ 40 or 42 at 100 µM in 10 mM Tris-HCl (pH 7.4) were centrifuged at 60,000 rpm for 72 hr at 4 °C using an An-60Ti rotor. The moving boundary was monitored continuously by the absorption at 230 nm with scanning recorder every 4 min. Sedimentation velocity data were analyzed using C(S) distribution method by SEDFIT (NIH, Bethesda, MD, USA) and the parameters were calculated using SEDNTERP (NIH, Bethesda, MD, USA).

### Solid-state NMR

Uniformly ^13^C labeled Aβ40 peptides were prepared by bacterial expression following our previous literature^93^. Briefly, the recombinant Aβ with His tag were prepared in minimal media in the transformed BL21(DE3) *E*. *coli* cells. The His-Aβ was purified through two Ni-affinity columns coupled with TEV protease digestion to remove the tag. The selectively ^13^C labeled peptides were synthesized on an automated Odyssey microwave peptide synthesizer (CEM Corp., Matthews, NC), using a FMOC-Valine-preloaded CLEAR-Acid resin (Peptide International) with 0.44 mequiv/g substitution level, and FMOC chemistry with hydroxybenzotriazole/N, N′-diisopropylcarbodiimide activation. The final products were validated by MALDI-TOF mass spectrometry. Aβ were incubated with equimolar of ZnCl_2_ for overnight. All NMR experiments were carried out carried out at ^13^C and ^1^H frequencies of 201.2 and 800.2 MHz, respectively, on a Bruker Avance III spectrometer equipped with a commercial 3.2 mm E-free probe. ^13^C chemical shifts were externally referenced to tetramethylsilane (TMS) using adamantane as the secondary reference. Spinning frequency at the magic angle was 10 kHz and the sample temperature was maintained at 268 K.

### Aβ seeding assay

Fifty µM Aβ fibril were prepared after shaking at 750 rpm for 5 days at room temperature. The fibril morphology was confirmed by TEM. The fibrils were sonicated in water bath for 10 mins, ZnAβ oligomers (filtered prior to use) and respective buffer were added to Aβ solution in all experiments was 5% (2.5 µM) of the concentration of the Aβ solution. The Aβ solution in the presence of 5 µM ThT were monitored in 384 well plates by ThT assay.

### Cytotoxicity assay

MTT assay was aimed to investigate the effect of Aβ on cell viability. The human neuroblastoma BE(2)-C cells (ATCC #CRL-2268) were cultured at 37 °C, 5% CO_2_ in the RPMI medium containing 10% FBS. Twenty thousand cells were seeding in a 96-well plate each well. ZnAβ or ADDLs diluted in the RPMI medium were added into the well and incubated for 24 hr. Then, ten µl MTT solution (5 mg/ml) was added and incubated for 3 hr for formation of formazan crystals. After incubation, the medium was carefully discarded and 100 µl DMSO was added to lyse the formazan crystals. Absorbance at 570 and 690 nm (background absorbance) were monitored by an ELISA microplate reader SpectraMax M5 (Molecular Devices, Sunnyvale, CA, USA). The difference of absorbance between 570 and 690 nm was calculated. For LDH assay, the assay was followed the instructions from the protocol of LDH assay toxicity kit (Promega, Madison, WI, USA). BE(2)-C cells were seeding the same as MTT ZnAβ or ADDLs were added and incubated for 24 hr. Next day, LDH assay was performed by monitoring the rate of substrate formation, emission at 590 nm while excitation at 560 nm. The results were replicated for 3 times. The cell lysed with 2% Triton X-100 was served as positive control for 100% cytotoxicity.

### Calcium influx assay

The fluorescence probe Fluo-3 AM (Invitrogen) was used to detect the intracellular Ca^2+^ levels. Twenty thousand BE(2)-C cells were seeded in a black 96-well plate with clear bottom. Next day, fluo-3 AM powder dissolved in DMSO was added into culture medium without FBS. The final concentration of 10 µM Fluo-3 AM was incubated for 1 hr within plated cell. The probe-containing medium was replaced by RPMI medium, then incubated at 37 °C for 10 mins, and fluorescence intensity was started to monitor prior to the addition of Aβ samples as stable baseline, and the wavelength of the fluorescence of Fluo-3 AM was excited at 480 nm and recorded emission wavelength at 560 nm. Data were averaged (n = 3).

### Animal

All experiments were done in accordance with the National Institutes of Health Guideline for Animal Research (Guide for the Care and Use of Laboratory Animals) and Taiwan Animal Protection Law and were approved by the Academia Sinica Institutional Animal Care and Utilization Committee (IACUC 12–03–340). All of the mice were housed (4–5 per cage) at a stable temperature (23 ± 1 °C), humidity 55 ± 5%, and unrestricted access to food and water during the experimental period.

### *In vitro* electrophysiology

Eight-week-old C57BL/6JNarl male mice were purchased from the National Laboratorial Animal Center and keep at the Animal Facility of the Genomic Research Center (GRC) at Academia Sinica. The mice were housed in GRC animal facility and following the GRC facility housing rule during the period. Animals were housed in a room maintained on a 12-h/12-h light/dark cycle (light on at 8:00 a.m.). Two to four-month-old C57BL/6 J mice were used to examine effect of ZnAβ42 oligomer. Mice were anaesthetized by isoflurane and decapitated. The brain was rapidly removed and immersed in ice-cold ACSF (which contained 119 mM NaCl, 2.5 mM KCl, 1.3 mM MgSO_4_, 2.5 mM CaCl_2_, 26.2 mM NaHCO_3_, 11 mM Glucose and 1.25 mM NaH_2_PO_4_). Cronol sections (450 μm) of hippocampal slices were immediately transferred to a chamber containing ACSF and 100 µM picrotoxin. Circulating ACSF was continuously bubbled with a mixture of 95% O_2_ and 5% CO_2_ and slices allowed to recover for at least 2 hr at room temperature. For extracellular recordings, the hippocampal regions were placed at the center of an MED–P515A probe (Panasonic International Inc.) with 64 embedded recording electrodes. The slices were randomly assigned to control or ZnAβ42 or Zn buffer treatment and subsequently perfused with ZnAβ42 at 500 nM in oxygenated ACSF for 0.5 hr before LTP induction. fEPSPs were recorded using a MED64 multichannel recording system, and the data were collected from Schaffer collaterals. Test stimuli were given every 30 s (0.033 Hz), and the stimulus intensity was set to give a baseline fEPSP of 10–90% of the maximal response. A stable baseline was recorded for at least 20 min. Filtered ZnAβ42 oligomer (stock solutions were diluted into ACSF to produce concentrations of 500 nM based on the starting weight of Aβ42 monomer), the sample was perfused for 30 mins prior to induction of LTP. LTP was induced by theta burst stimulation (10 bursts of 4 stimuli at 100 Hz, with an interburst interval of 200 ms) given at baseline intensity according to previously described^74^. The ACSF was recycled using peristaltic pumps ensuring that the ZnAβ42 was present for the duration of the experiment. LTP is expressed as the mean ± SEM % of baseline fEPSP slope. Statistical comparisons used two-way ANOVA with post hoc Bonferroni test. In addition, control/ZnAβ42/Zn^2+^ buffer was performed on the same day to avoid any temporal bias.

### Acute Aβ-injected mice models

The experiment procedure of acute Aβ-injected-models was followed and modified from previous protocols^94–98^. Eight-week-old C57BL/6 JNarl male mice were first intraperitoneally anesthetized by zoletil^TM^ (100 mg/kg) plus xylazine hydrochloride (10 mg/ kg, Sigma-Aldrich, St. Louis, MO, USA). Forty µM filtered ZnAβ42/ADDLs oligomer or their respective buffer was bilaterally injected into the hippocampus at following stereotaxic coordinated: 2 mm posterior to the bregma, 2.1 mm left/right to the midline, and 1.8 mm ventral to the skull surface. The volume of injection was 2 µl and lasted for 5 mins, and remain in injected region for 5 mins followed by injection to avoid Aβ reflux along the needle tract, 14 days were allowed for the development of Aβ-induced damage.

### Immunohistochemistry

Prior to processing immunohistochemistry, mice were anesthetized and perfused with 0.9% ice cold NaCl. After removing the brains, left hemispheres were stored at −80 °C, and the right hemispheres were subjected to fixed in 10% neutral buffered formalin solution (Sigma-Aldrich, St. Louis, MO, USA) at 4 °C for 24 hrs, dehydrated and cyroprotected in 30% sucrose solution then embedded in OCT (Leica, Mannheim, Germany). The brains were further cut-into 25 µm-thick coronal sections on a cryostat (Leica CM3050S, Mannheim, Germany). The sections were washed in PBS and mounted on poly-L lysine-coated slides (Thermo Fisher Scientific, Waltham, MA, USA), dried overnight. Then, the coronal sections were washed, antigen retrieval heated at 80 °C in sodium citrate buffer for 30 mins, washed, treated with 1% H_2_O_2_ to inactivate endogenous peroxidase activity, blocking in 3% BSA for 30 mins. The sections were further stained with following primary antibodies: anti-Iba1 (1:1000, Millipore, Temecula, CA, USA) for microglia. To analyze protein levels, images were captured by Aperio AT2 Digital Pathology Scanner (Leica Biosystem, Mannheim, Germany). For microglia activation, we selected coronal sections 360 μm apart between bregma −1.34 mm to −3.80 mm, which encompassed hippocampal region and further analyzed by Image J (NIH, Bethesda, MD, USA). To quantify density and surface area of microglia and astrocyte, we first optimized brightness and contrast of control group, then manually set the best threshold value. The area of Iba1^+^ was measured with the signal which was higher than background. For the density, cells with clear and identifiable cell bodies were counted. The densities were represented by dividing cell numbers by the area of the hippocampal region.

### Data Availability

The datasets generated during and/or analyzed during the current study are available from the corresponding author on reasonable request.

## Electronic supplementary material

Supplementary information
