# Supplementary material for: Zinc ion rapidly induces toxic, off-pathway amyloid-β oligomers distinct from amyloid-β derived diffusible ligands in Alzheimer’s disease
Source: Sci Rep. 2018 Mar 19;8:4772. doi: 10.1038/s41598-018-23122-x (PMC5859292; doi:10.1038/s41598-018-23122-x)

Supporting Information

Zinc ion rapidly induces toxic, off-pathway amyloid-β oligomers distinct from amyloid-β derived diffusible ligands in Alzheimer's disease

Ming-Che Lee[1,2], Wan-Cheng Yu[3], Yao-Hsiang Shih[2], Chun-Yu Chen[2], Zhong-Hong Guo[3], Shing-Jong Huang[3], Jerry C. C. Chan[3], and Yun-Ru Chen[1,2*]

Supplementary Method

ThT assay. ThT assays of Aβ40 and Aβ42 at 50 μM were performed in the presence of 0, 10, 25, 50, 100 and 200 μM Zn^{2+} according to the method. The final ThT concentration was 5 μM. ThT fluorescence was monitored at 25 °C.

Turbidity assay. Turbidity was measured at absorbance 340 nm by an UV/Vis spectrophotometer DU800 (Beckman Coulter, CA). The data were normalized with absorbance of Aβ40 or Aβ42 on day 0. The experiment was repeated three times and the data were averaged and presented as mean ± S.D.

Diameter distribution of oligomers. TEM images of ZnAβ or ADDLs were acquired from FEG-TEM. The diameter distribution was calculated by software Image J (NIH, Bethesda, MD, USA). Size distribution was calculated from at least 60 oligomers of ZnAβ or ADDLs.

Zn^{2+} treatment on Aβ fibrils. Aβ solution at 50 μM was prepared with 5 μM ThT. The samples were incubated at 25 °C with agitation for 60 sec every hr. After ThT fluorescence reached maximum intensity, equal molar concentration of $ZnCl_2$ or buffer was added into fibril solution and ThT intensity was monitored.

FTIR. FTIR spectra were recorded with a Thermo Scientific Nicolet™ 6700 spectrometer equipped with an ATR sampling accessory (PIKE MIRacle, USA). Fifty μl Aβ fibrils were deposited onto ATR crystal, and the solvent was evaporated by an air dryer blowing air in room temperature. Spectra were recorded at a resolution of 2

cm^{-1} and accumulated for 100 times at a wave number range from 900 to 4,000 cm^{-1}. The signals were calculated and subtracted from buffer control. Then, the signals were normalized with the individual peak in maximal intensity.

Dot blotting. Two µl of the each Aβ time-course samples were collected at a series of time points and dotted onto nitrocellulose membrane with several duplicates for probing by different antibodies. Membranes were probed with A11 (Invitrogen), OC (Millipore, Temecula, CA, USA), 4G8 (COVANCE, NJ, USA), 6E10 (COVANCE, NJ, USA), Aβ 22-35 (Sigma-Aldrich, St. Louis, MO, USA), C-terminus of Aβ42 (Millipore) separately. Membrane was also staining with direct blue as a loading control to check equal loading of each dot. The seperate blots were shown in groups with dividing lines in the figures.

Solid-state NMR. See method in text.

Immunohistochemistry. For astrogliosis staining, the sections were stained with anti-GFAP (1:1000, GeneTex, Irvine, CA, USA) for astrocytes. We selected coronal sections 360 µm apart from bregma -1.34 mm to -3.80 mm, which encompassed hippocampal region and further analyzed by Image J (NIH, Bethesda, MD, USA). To quantify density and surface area of astrocyte, we first optimized brightness and contrast of control group, then manually set the best threshold value. The area of $GFAP^{+}$ were measured with the signal which was higher than the background. For density, cells with clear and identifiable cell bodies were counted. The densities were represented by dividing cell numbers by the area of the hippocampal region.

Supplementary Figure legends

Supplementary Figure 1. Effect of various Zn^{2+} concentrations on Aβ fibrillization by ThT assay. ThT assays of (A) Aβ40 and (B) Aβ42 at 50 µM were performed in the presence of 0, 10, 25, 50, 100 and 200 µM Zn^{2+}. The final ThT concentration was 5 µM. ThT fluorescence was monitored at 25 °C.

Supplementary Figure 2. Turbidity of Aβ in the presence of Zn^{2+}. Turbidity indicated by absorbance 340 nm was measured at different time points with 50 µM (A) Aβ40 and ZnAβ40 and (B) Aβ42 and ZnAβ42. The protein concentrations of (C) Aβ40 and ZnAβ40 and (D) Aβ42 and ZnAβ42 were also measured at 280 nm. Data was normalized with absorbance of freshly-prepared Aβ at day 0. Data was shown as mean ± S.D.

Supplementary Figure 3. TEM images of ZnAβ and the diameter distribution. TEM images of (A) ZnAβ40 and (B) ZnAβ42. The scale bars are 50 nm. The diameter distribution was calculated from TEM images of (C) ZnAβ40 and (D) ZnAβ42 by Image J. The distribution is calculated from 60 oligomers.

Supplementary Figure 4. Preformed Aβ fibrils is not affected by Zn^{2+}. (A) ThT assay of Aβ40 with addition of Zn^{2+} after fibril formation. Aβ40 was incubated for fibril formation until ThT signal reached a plateau phase. Then, equal molar concentration of Zn^{2+} or its buffer was added at ~120 hr of incubation time as indicated by arrow. (B) FTIR spectra of Aβ40 fibrils treated with Zn^{2+} or its buffer. No significant difference was observed after treatment.

Supplementary Figure 5. ZnAβ oligomers show lower immunoreactivity to various anti-Aβ antibodies. (A-B) Dot blotting of time-course samples during Aβ aggregation were collected from Aβ40 (A) and Aβ42 (B) with and without Zn^{2+}. The samples were probed with anti-oligomer antibody A11, anti-fibril antibody OC, anti-Aβ residues 1–16 antibody 6E10, anti-Aβ residues 17–24 antibody 4G8 as indicated. The membranes were also stained with Direct blue to indicate equal sample dotting. (C-D) Dot blotting of sample was collected from ADDL40 and ZnAβ40 (C) as well as ADDL42 and ZnAβ42 (D) incubated for 1 or 7 days. Membranes were probed with anti-oligomer antibody A11, anti-fibril antibody OC, anti- Aβ residues 1–16 antibody

6E10, anti- Aβ residues 17–24 antibody 4G8, anti-Aβ residues 22–35 antibody A3356, or anti- Aβ42 residues 36–42 antibody separately as indicated. The separated blots were shown in groups with dividing lines.

Supplementary Figure 6. ^{13}C homonuclear correlation spectrum acquired for the ^{13}C uniformly labeled ZnAβ40. The resolution of the spectrum did not allow the site-specific assignment of the signals.

Supplementary Figure 7. ^{13}C correlation spectrum and the spectral assignment of site-specific labeled ZnAβ40.

Supplementary Figure 8. Effect of ZnAβ42 oligomer on hippocampal astrocyte activation in the wild-type mice. (A) The micrograph represents the GFAP^{+} positive microglia in the hippocampus of the mice after ZnAβ42 or ADDLs injection. Quantitative results of GFAP^{+} microglia are represented as density (B, D) and area (%) (C, E) (ZnAβ42, n=19, Zn buffer, n=23, ADDL42, n=24, ADDL buffer, n=17, and n values are from the brain slices of 3 mice of each group. Scale bar = 800 μm).

Supplementary Figure 1

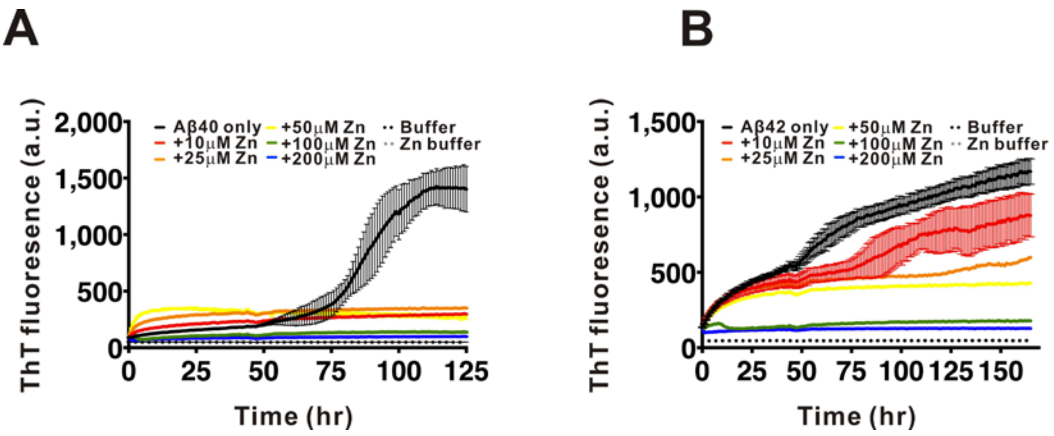

Supplementary Figure 2

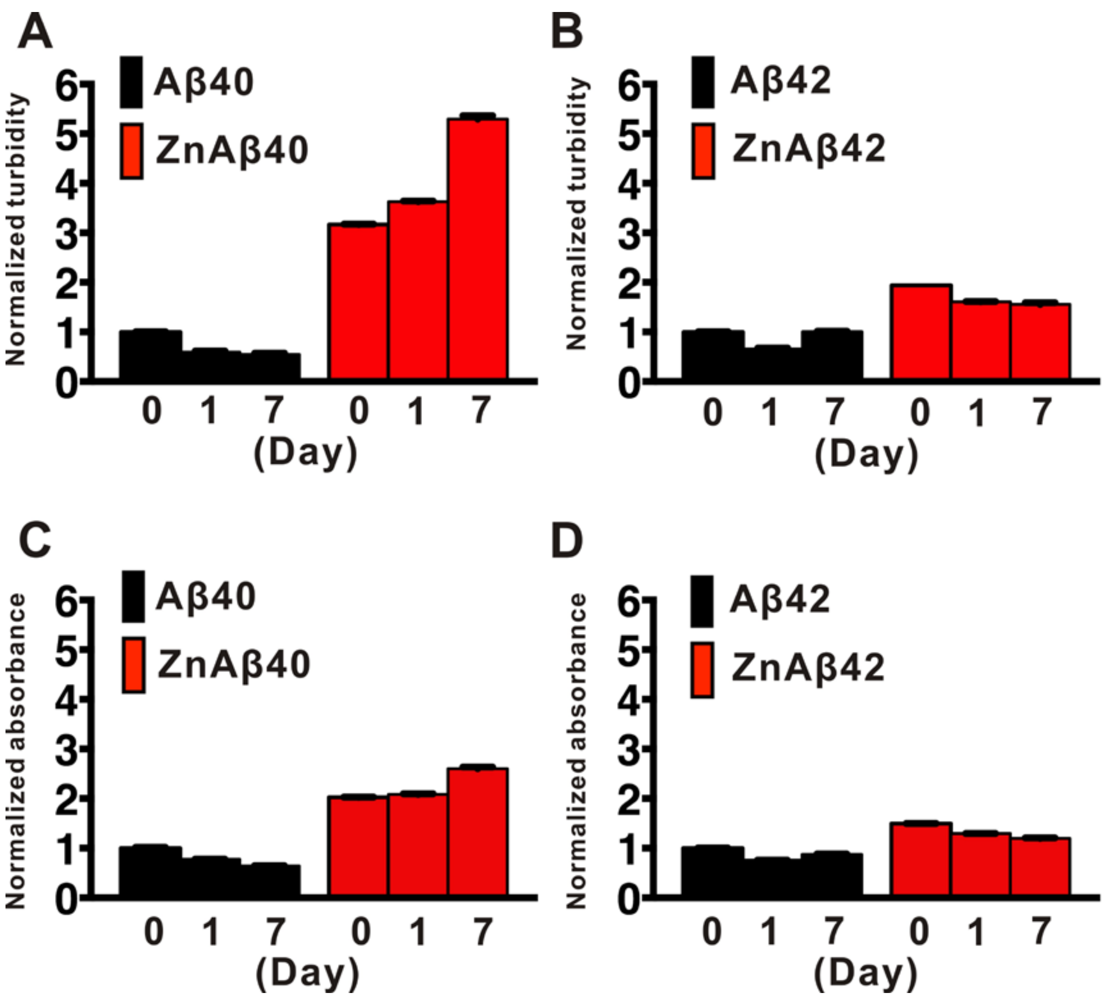

Supplementary Figure 3

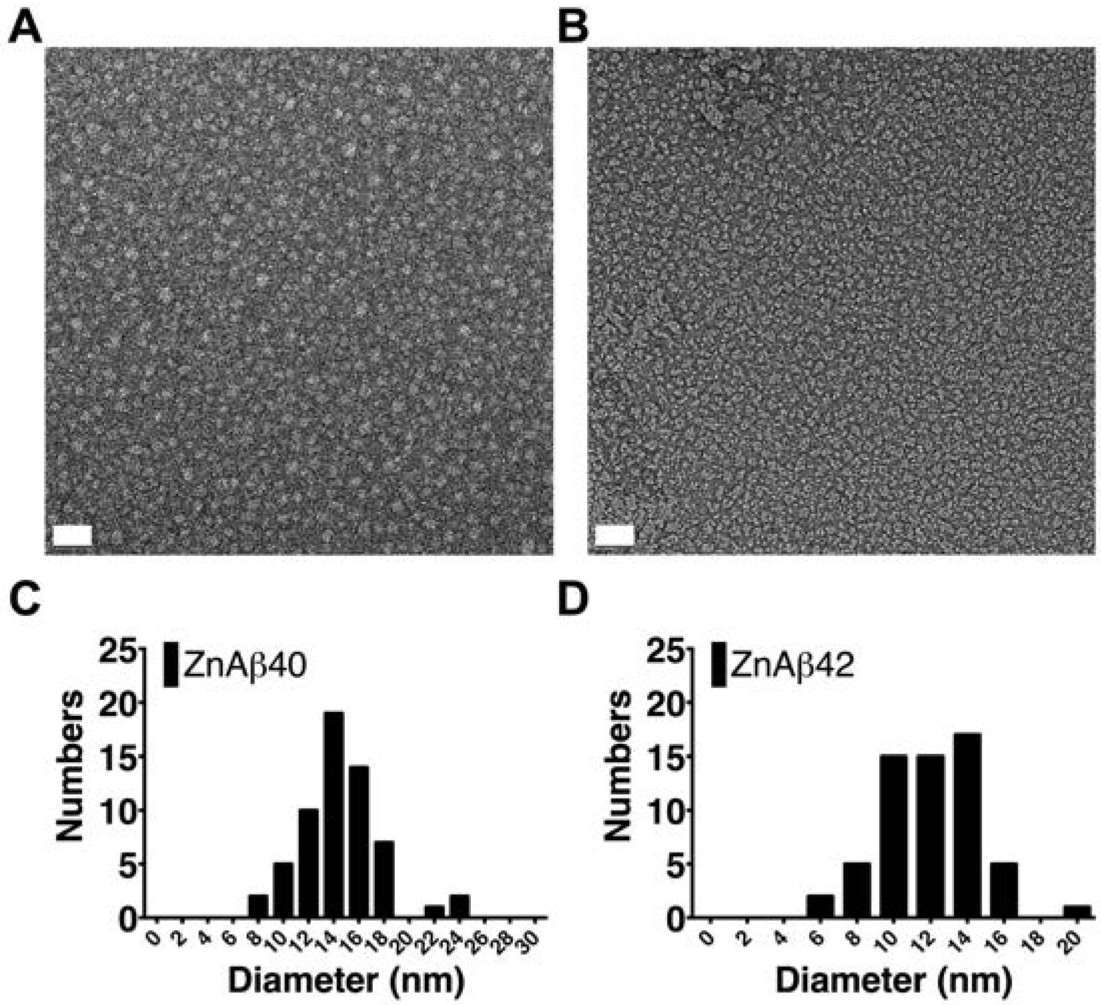

Supplementary Figure 4

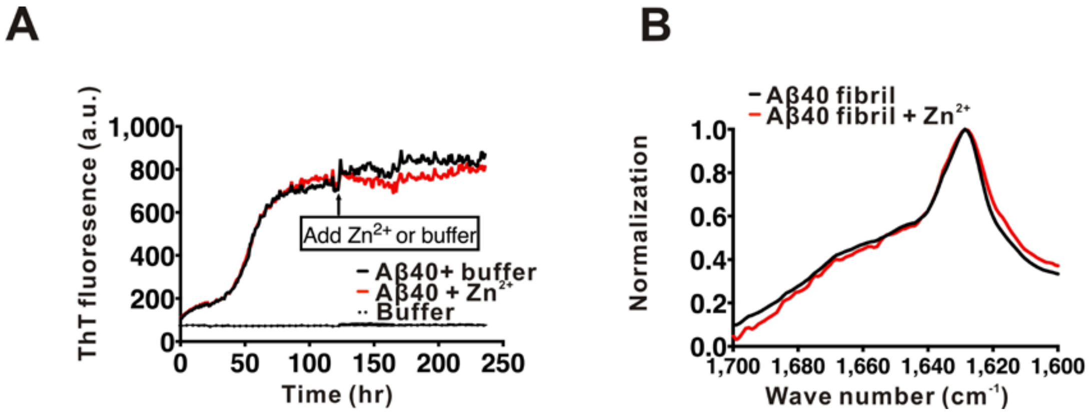

Supplementary Figure 5

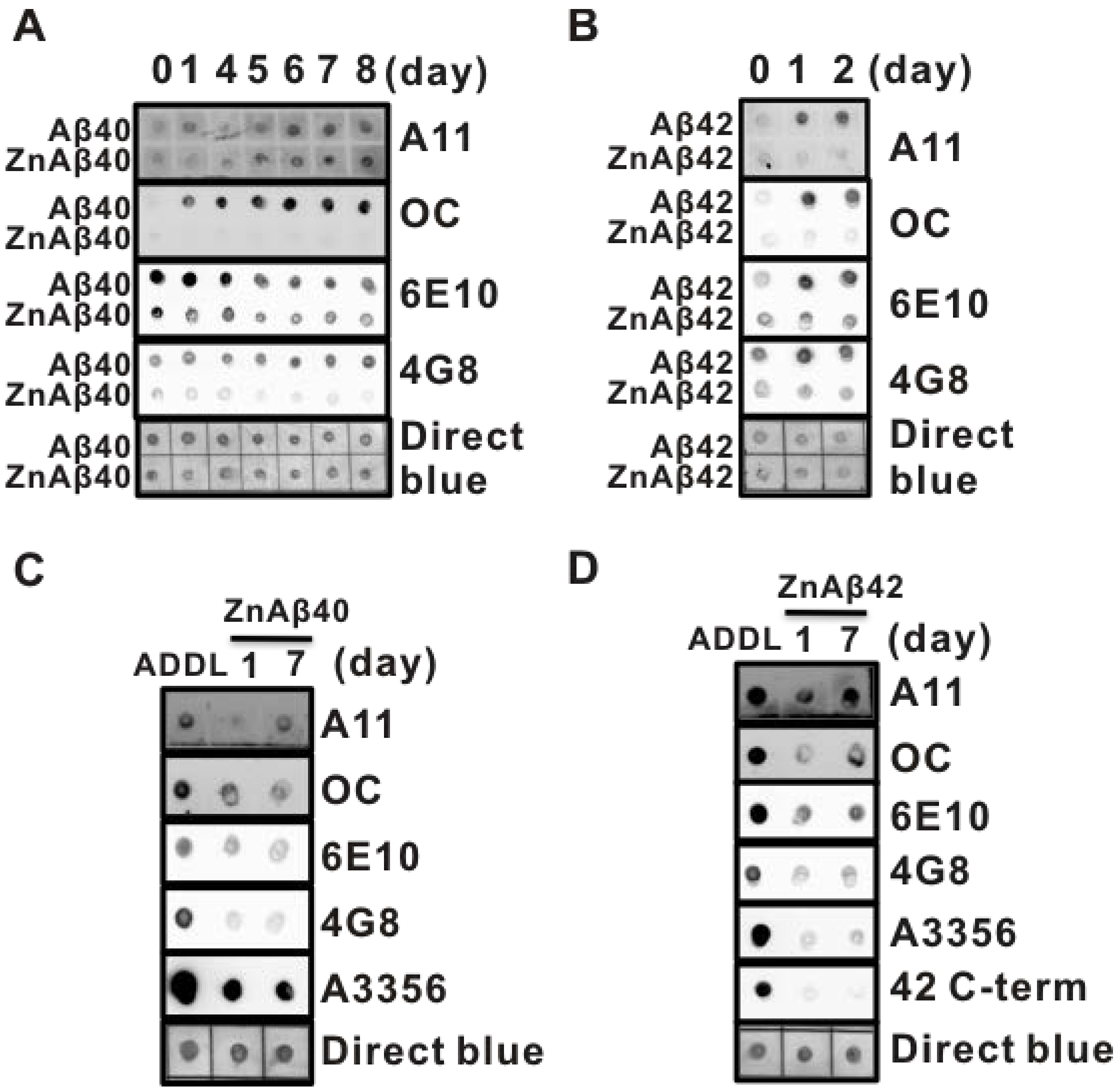

Supplementary Figure 6

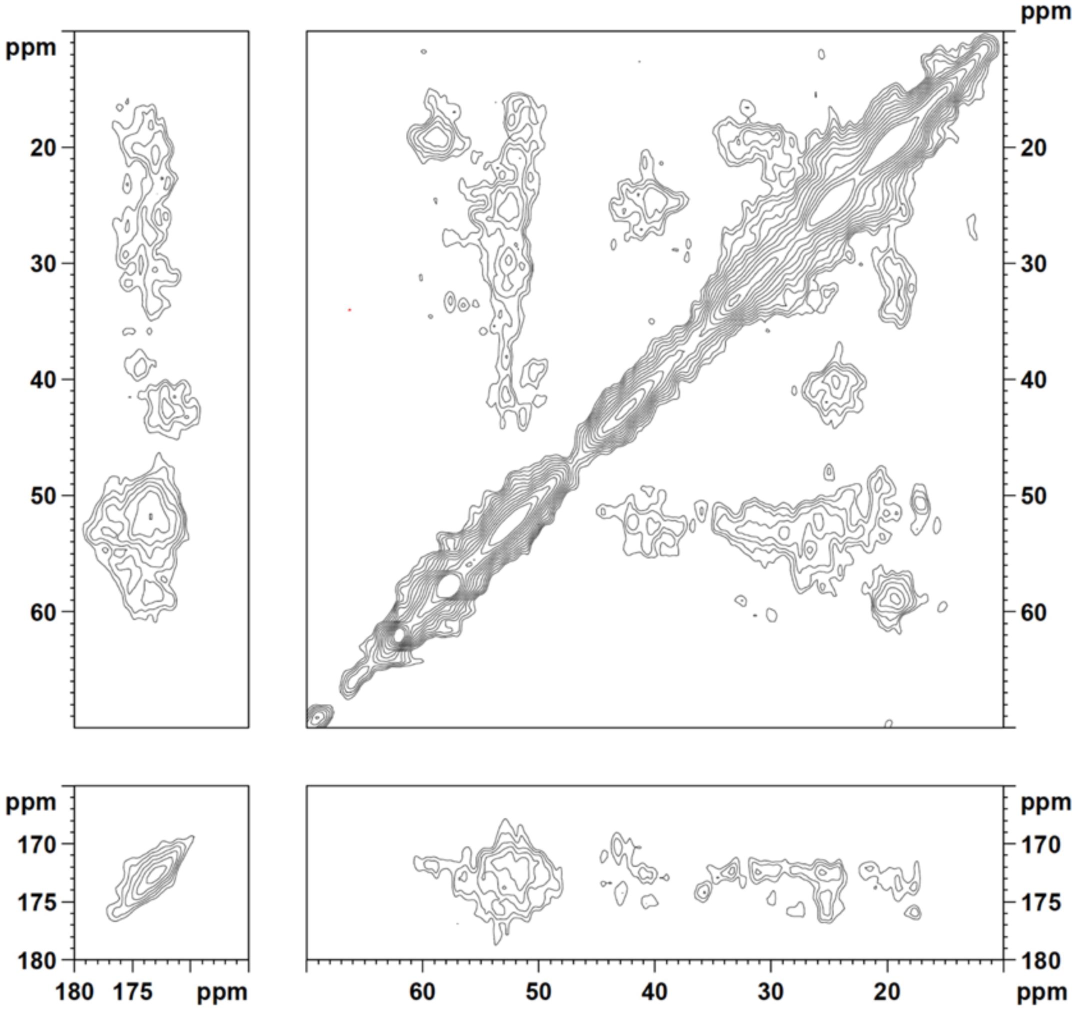

Supplementary Figure 7

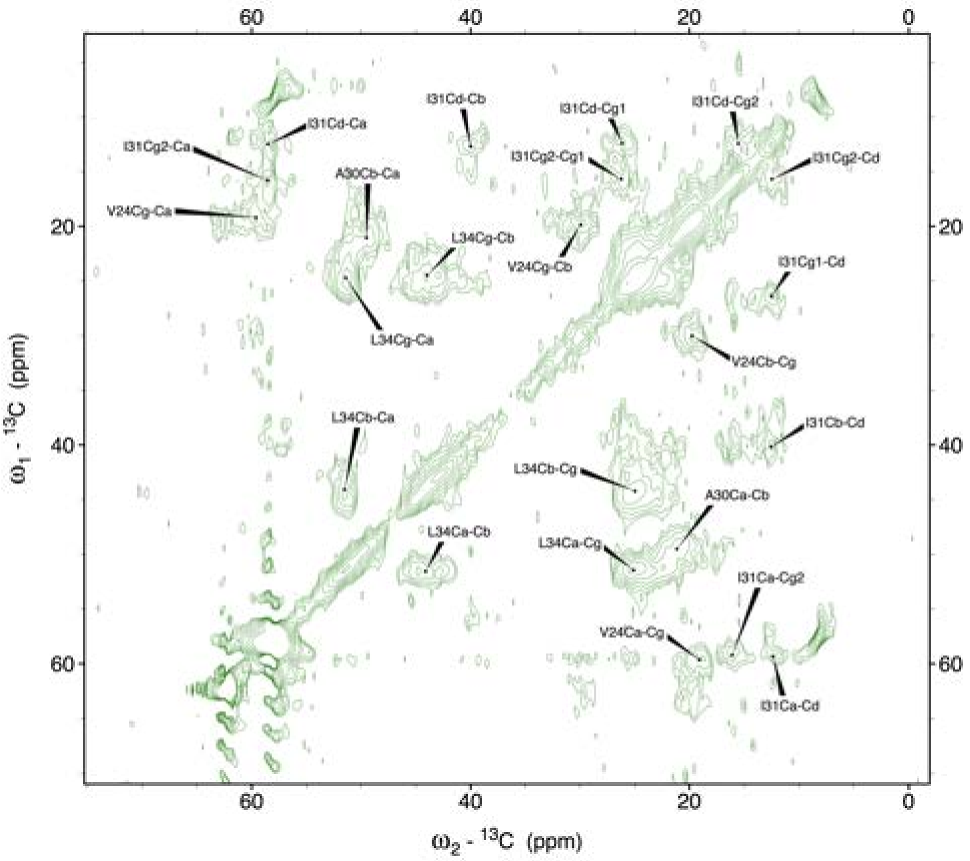

Supplementary Figure 8

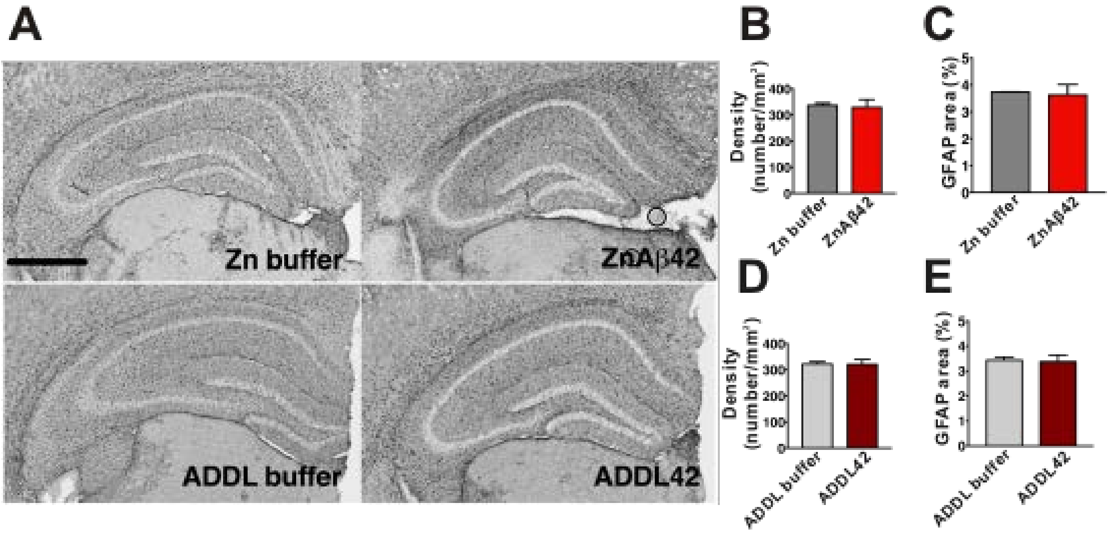

Supplement: Supplementary file 1 — Supplementary information [file 41598_2018_23122_MOESM1_ESM.pdf]
